# The long non-coding RNA *SAMMSON* is essential for uveal melanoma cell survival

**DOI:** 10.1038/s41388-021-02006-x

**Published:** 2021-09-10

**Authors:** Shanna Dewaele, Louis Delhaye, Boel De Paepe, Eric James de Bony, Jilke De Wilde, Katrien Vanderheyden, Jasper Anckaert, Nurten Yigit, Justine Nuytens, Eveline Vanden Eynde, Joél Smet, Maxime Verschoore, Fariba Nemati, Didier Decaudin, Manuel Rodrigues, Peihua Zhao, Aart Jochemsen, Eleonora Leucci, Jo Vandesompele, Jo Van Dorpe, Jean-Christophe Marine, Rudy Van Coster, Sven Eyckerman, Pieter Mestdagh

**Affiliations:** 1 OncoRNALab, Center for Medical Genetics (CMGG), Ghent University, Ghent, Belgium; 2 Cancer Research Institute Ghent (CRIG), Ghent, Belgium; 3 Department of Biomolecular Medicine, Ghent University, Ghent, Belgium; 4 Center for Medical Biotechnology, VIB-Ghent University, Ghent, Belgium; 5 Department of Pediatrics, Division of Pediatric Neurology and Metabolism, Ghent University Hospital, Ghent, Belgium; 6 Department of pathology, Ghent University Hospital, Ghent, Belgium; 7 Institut Curie, Laboratory of Preclinical Investigation, Translational Research Department, PSL Research University, Paris, France; 8 Institut Curie, Department of Medical Oncology, PSL Research University, Paris, France; 9 Inserm U830, DNA Repair and Uveal Melanoma (D.R.U.M.), Equipe labellisée par la Ligue Nationale Contre le Cancer, Institut Curie, PSL Research University, Paris, 75005 France; 10 Center for Medical Biotechnology, VIB-KU Leuven Center for Cancer Biology, Leuven, Belgium; 11 Laboratory for RNA Cancer Biology, Department of Oncology, KU Leuven, Leuven, Belgium; 12 Department of Cell and Chemical Biology, Leiden University Medical Center, Leiden, The Netherlands; 13 TRACE, LKI Leuven Cancer Institute, Leuven, Belgium; 14 Laboratory for Molecular Cancer Biology, Center for Cancer Biology, VIB, Leuven, Belgium; 15 Laboratory for Molecular Cancer Biology, Department of Oncology, KULeuven, Leuven, Belgium

**Keywords:** Targeted therapies, Non-coding RNAs

## Abstract

Long non-coding RNAs (lncRNAs) can exhibit cell-type and cancer-type specific expression profiles, making them highly attractive as therapeutic targets. Pan-cancer RNA sequencing data revealed broad expression of the *SAMMSON* lncRNA in uveal melanoma (UM), the most common primary intraocular malignancy in adults. Currently, there are no effective treatments for UM patients with metastatic disease, resulting in a median survival time of 6–12 months. We aimed to investigate the therapeutic potential of *SAMMSON* inhibition in UM. Antisense oligonucleotide (ASO)-mediated *SAMMSON* inhibition impaired the growth and viability of a genetically diverse panel of uveal melanoma cell lines. These effects were accompanied by an induction of apoptosis and were recapitulated in two uveal melanoma patient derived xenograft (PDX) models through subcutaneous ASO delivery. *SAMMSON* pulldown revealed several candidate interaction partners, including various proteins involved in mitochondrial translation. Consequently, inhibition of *SAMMSON* impaired global, mitochondrial and cytosolic protein translation levels and mitochondrial function in uveal melanoma cells. The present study demonstrates that *SAMMSON* expression is essential for uveal melanoma cell survival. ASO-mediated silencing of *SAMMSON* may provide an effective treatment strategy to treat primary and metastatic uveal melanoma patients.

## Introduction

Uveal melanoma (UM) is the most common primary intraocular malignancy in adults, with an incidence of 5−7.4 cases per million annually [1–3]. Current treatments consist of radiotherapy and enucleation, but despite the advances in local therapy towards eye-preserving therapeutic choices, no substantial progress in overall survival has been achieved. The main cause of death of UM patients is due to the metastatic dissemination, mainly to the liver, in about 50% of the patients. Patients with metastatic UM have extremely poor survival, with a median survival time of 6−12 months [4]. Uveal melanoma is a genetically and biologically distinct type of melanoma that arises from choroidal melanocytes in the choroidal plexus, ciliary body and iris of the eye. In addition, UM and cutaneous melanoma differ in their chromosomal aberrations and mutational signature. Recurrent genomic aberrations in UM include loss of 1p, monosomy of chromosome 3, loss of 6q and 8p and gain of 6p and 8q, of which loss of chromosome 3 and gain of 8q have been associated with a high mortality rate [5, 6]. Tumor cells are characterized by activated G protein-coupled receptor (GPCR) signaling, which occurs in almost all UM tumors by specific mutations that have been related to UM, such as mutations in *GNAQ or GNA11* (90% of UM tumors). These mutations result in the activation of the pathways downstream of Gα_q_ and Gα_11_ such as the Ras Homolog Family Member/Ras-related C3 botulinum toxin/Yes Associated Protein (Rho/Rac/YAP) pathway, Phosphatidylinositol (4,5)-bisphosphate 3-kinase (PI3K)/AKT pathway and Phospholipase C (PLC) which subsequently activates the Mitogen-Activated Protein Kinase (MAPK) pathway (RAF/MEK/ERK) [7]. Although *GNAQ* and *GNA11* mutations occur in most UM, they are not associated with metastasis [8]. In addition, inactivating somatic mutations in BRCA1-associated protein 1 (*BAP1*) have been frequently detected in UM tumors and are associated with the development of metastasis [9]. Tumor suppressor gene *BAP1*, located on 3p21.1, is frequently mutated on the remaining allele present in monosomy 3 tumors, which might explain the poor prognosis of monosomy 3 tumors [10]. Loss of BAP1 affects several pathways, including DNA damage repair, cell cycle regulation, cell differentiation and cell growth [11, 12]

Long non-coding RNAs are an emerging class of regulatory RNA molecules that interact with proteins, DNA, and other RNA molecules. Compared to protein-coding mRNAs, lncRNAs show a more tissue restricted expression profile, making them attractive targets for therapy. LncRNAs are regulating a variety of cellular functions such as transcription, splicing, mRNA stability and translation, and do so via different mechanisms. Recently, Survival Associated Mitochondrial Melanoma Specific Oncogenic Non-coding RNA (*SAMMSON*) was discovered on chromosome 3p13 as a lineage survival oncogene in skin melanoma [13]. Interaction of *SAMMSON* with proteins involved in ribosomal RNA (rRNA) maturation and protein synthesis such as p32 (C1QBP), CARF and XRN2 and sequestration of CARF in the cytoplasm result in an elevated cytosolic and mitochondrial rRNA processing and protein synthesis [14]. In this study, we show that *SAMMSON* is consistently expressed in UM and conjunctival melanoma (CM) cells, the latter of which are phenotypically and genetically more related to skin melanoma. *SAMMSON* silencing, by means of locked nucleid acid (LNA) antisense oligonucleotides (ASOs), revealed an essential role for *SAMMSON* in UM survival in vitro and in vivo and provides perspectives for RNA-targeted therapy.

## Results

### *SAMMSON* is consistently expressed in uveal melanoma tumors

**Fig. 1:**
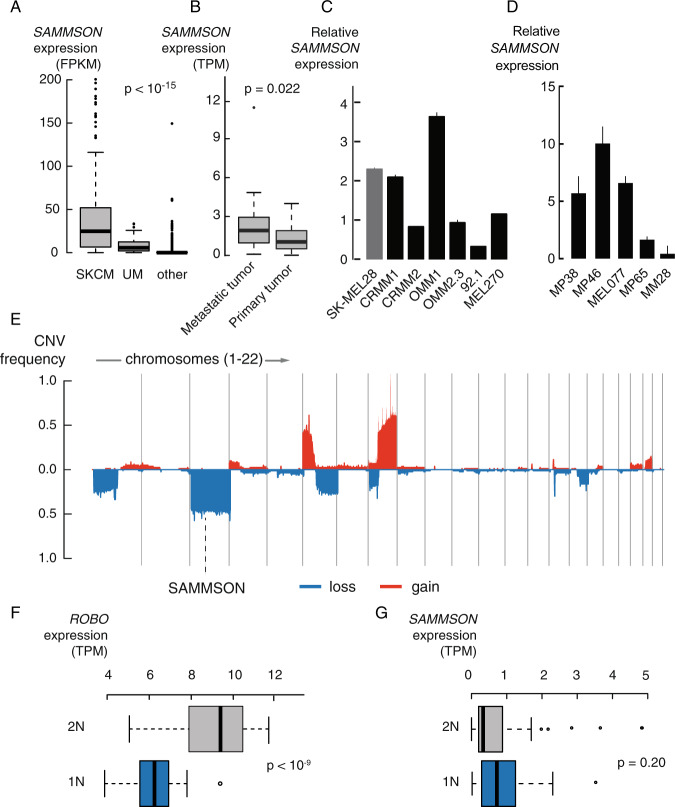
LncRNA *SAMMSON* is consistently expressed in UM tumors. **A** RNA sequencing data from >10,000 tumor samples and 32 cancer types (TCGA) showing *SAMMSON* expression in skin melanoma (SKCM), uveal melanoma (UM) and other cancer types (*p* < 10^−15^, Mann–Whitney test). **B** RNA sequencing data showing elevated *SAMMSON* expression levels in metastatic UM tumors compared to matching primary tumors (*n* = 21, *p* = 0.022, Wilcoxon matched-pairs signed rank test). **C** Relative *SAMMSON* expression in CM cell lines (CRMM1, CRMM2) and UM cell lines (OMM1, OMM2.3, 92.1 and MEL270) compared to skin melanoma cell line SK-MEL28. Error bars represent ± standard error (SE) of qPCR replicates. **D** Relative *SAMMSON* expression in UM PDX lines. Error bars represent ± standard error (SE) of qPCR replicates. **E** Frequent chromosomal aberrations in UM tumors and the location of *SAMMSON* on chromosome 3, which is frequently lost. **F** Significantly lower *ROBO1* expression levels in monosomy 3 (1 N, *n* = 36) tumors compared to disomy 3 (2 N, *n* = 44) tumors (*p* < 10^−9^, Mann–Whitney test). **G** No difference in *SAMMSON* expression levels between disomy 3 (2 N, *n* = 44) and monosomy 3 (1 N, *n* = 36) tumors (*p* = 0.20, Mann–Whitney test).

Pan cancer RNA sequencing data from more than 10 000 tumor samples representing 32 cancer types (The Cancer Genome Atlas, TCGA) showed the highest and most consistent *SAMMSON* expression in skin melanoma (SKCM, *SAMMSON* expression in >90% of tumor samples) followed by uveal melanoma (*SAMMSON* expression in >80% of tumor samples) (Fig. 1A, *p* < 10^−15^, Mann–Whitney test). While *SAMMSON* expression in uveal melanoma tumors is independent from patient survival, tumor stage, primary tumor localization site (choroid, ciliary body or iris) and metastatic stage of the patient, *SAMMSON* expression is elevated in metastatic tumors compared to matched primary tumors (Fig. 1B, *p* = 0.022, Wilcoxon matched-pairs signed rank test, Supplemental Fig. 1). *SAMMSON* expression was further verified by RT-qPCR in various UM cell lines originating from primary tumors (92.1 and MEL270) and metastatic tumors (OMM2.3 and OMM1) (Fig. 1C), as well as in UM PDX-derived cell lines (MP38 (BAP1 negative, monosomy 3), MP46 (BAP1 negative, monosomy 3), MEL077, MP65 (BAP1 negative, monosomy 3), and MM28) (Fig. 1D). *SAMMSON* expression was also detected in the conjunctival melanoma (CM) cell lines CRMM1 and CRMM2 that are genetically and phenotypically more related to skin melanoma [15] (Fig. 1C). In contrast to skin melanoma tumors, where *SAMMSON* is invariably co-amplified with Melanocyte Inducing Transcription Factor *(MITF)* on chromosome 3, uveal melanoma cells are characterized by frequent (50−60%) loss of an entire copy of chromosome 3 (ref. [6, 16]) (Fig. 1E). While the majority of genes expressed from chromosome 3, for example Roundabout Guidance Receptor 1 *(ROBO1)* (Fig. 1F, *p* < 10^−9^, Mann-Whitney test), are significantly downregulated in monosomy 3 UM tumors (Supplemental Fig. 2, 89% downregulated genes (*n* = 581), 81% of those genes are significantly downregulated (*n* = 472/581)), *SAMMSON* expression is independent of chromosome 3 copy number (Fig. 1G, *p* = 0.20, Mann–Whitney test). This suggests a compensation mechanism by which UM tumors cells maintain high *SAMMSON* levels in the presence of chromosome 3 loss.

### *SAMMSON* expression is required for UM and CM cell survival in vitro

**Fig. 2:**
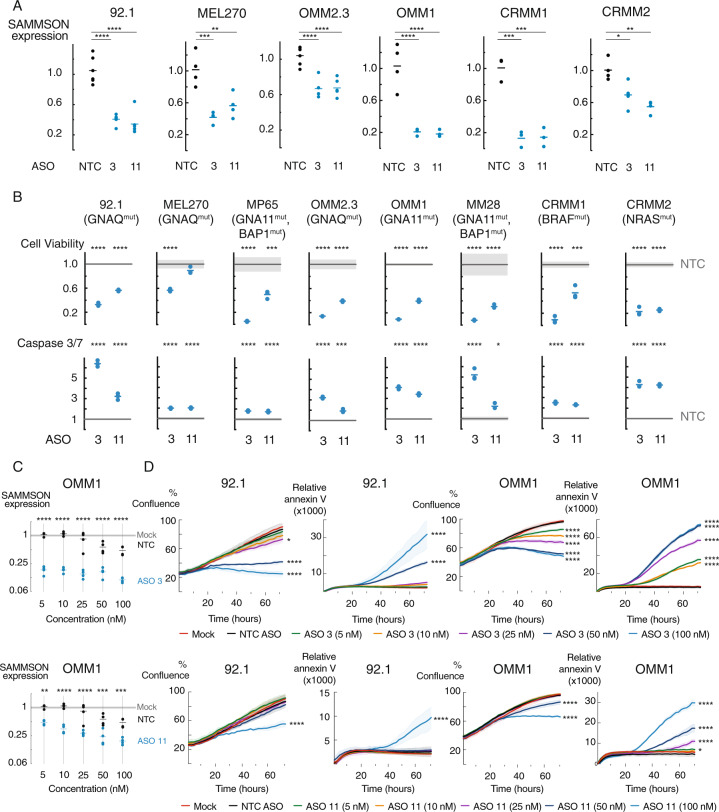
*SAMMSON* knockdown reduces cell viability and confluence and induces apoptosis. **A** Relative *SAMMSON* expression 48 h after transfection in four UM cell lines (92.1, MEL270, OMM2.3, OMM1) and two CM cell lines (CRMM1, CRMM2) transfected with 100 nM of a scrambled ASO (NTC), ASO 3 or ASO 11. The individual data points and mean are presented. **B** Cell viability (Cell Titer Glo) reduction and apoptosis (Caspase-Glo 3/7) induction in ASO 3 and ASO 11 treated UM and CM (CRMM1 and CRMM2) cells compared to NTC ASO treated cells (72 h post-transfection). The individual data points and mean of ASO 3 and ASO11 are presented. The NTC ASO treated data are presented as the mean of three replicates ± s.d. *P* values in (**A**) and (**B**) were calculated for each ASO 3/ASO 11 concentration and compared to NTC ASO using one-way ANOVA with Dunnett’s multiple testing correction. **C** Relative *SAMMSON* expression in UM cell line OMM1 48 h after transfection with 5, 10, 25, 50, or 100 nM of NTC ASO, ASO 3 or ASO 11 and scaled to untreated cells (Mock). The individual data points and mean of NTC ASO, ASO 3 and ASO11 are presented. The mock treated data are presented as the mean of four replicates ± s.d. *P* values were calculated for each ASO 3/ASO 11 concentration and compared to its corresponding NTC ASO concentration using one-way ANOVA with Dunnett’s multiple testing correction. **D** Reduction of proliferation (% confluence) and induction of apoptosis (annexin V) in 92.1 and OMM1 upon transfection with 5, 10, 25, 50, or 100 nM ASO 3 or ASO 11 compared to NTC ASO treatment or untreated cells (mock) measured by time-lapse microscopy (every 2–3 h) using the IncuCyte device. Data represents mean ± s.d. of three replicates. NTC ASO data represents the mean ± s.d. of all NTC ASO concentrations (5, 10, 25, 50, and 100 nM). *P* values were calculated at the 72 h time point compared to NTC ASO using one-way ANOVA with Dunnett’s multiple testing correction. *p ≤ 0.05, **p ≤ 0.01, ***p ≤ 0.001, ****p ≤ 0.0001.

To evaluate the importance of *SAMMSON* in UM and CM, we studied the effects of *SAMMSON* knockdown in various UM and CM cell lines using two independent *SAMMSON* targeting ASOs (ASO 3 and ASO 11). Both ASOs (ASO 3 i.e. GapmeR3 and ASO 11 i.e. GapmeR11) have previously been validated by Leucci et al. [13] for its efficient knockdown performance without toxic effects in *SAMMSON* negative cell lines. Transfection of 100 nM of both *SAMMSON* inhibiting ASOs significantly decreased *SAMMSON* expression in four UM (92.1, MEL270, OMM2.3 and OMM1) and two CM cell lines (CRMM1 and CRMM2) (Fig. 2A, *p* < 0.05, one-way ANOVA). *SAMMSON* knock-down resulted in a strong and significant decrease in cell viability (based on ATP measurements) in multiple UM and two CM cell lines (Fig. 2B, p≤0.001 for all except ASO 11 in MEL270, one-way ANOVA). These effects were accompanied by the induction of apoptosis, as evidenced by a significant increase in caspase-3/7 levels, observed for both *SAMMSON* inhibiting ASOs in 8 different UM and CM cell lines (Fig. 2B, *p* < 0.05, one-way ANOVA). In line with earlier observations in skin melanoma cells [13], these effects were independent of the mutational status (GNAQ, GNA11, BAP1, BRAF and NRAS) of the UM and CM cell lines.

To evaluate whether *SAMMSON* knockdown depends on ASO dosing, we performed a dose response experiment with both ASOs in the UM cell line OMM1 and CM cell line CRMM1. We observed a dose dependent decrease in *SAMMSON* expression for both ASOs (Fig. 2C and Supplemental Fig. 3A, *p* ≤ 0.0001 and *p* ≤ 0.001 for all tested concentrations of ASO 3 in OMM1 and CRMM1, respectively and *p* ≤ 0.01 for all tested concentrations of ASO 11 in OMM1 and CRMM1, except 5 nM in CRMM1, one-way ANOVA). We also observed a dose dependent reduction in cell growth and proliferation in UM cell lines OMM1, 92.1 and CM cell line CRMM1, measured via real-time cell imaging (Fig. 2D and Supplemental Fig. 3B, *p* < 0.05 for the three highest ASO 3 concentrations and the highest ASO 11 concentration in 92.1, *p* ≤ 0.0001 for all ASO 3 concentrations and the 2 highest ASO 11 concentrations in OMM1 and *p* ≤ 0.0001 for the three highest ASO three and two highest ASO 11 concentrations in CRMM1, one-way ANOVA). These effects were accompanied by a dose dependent induction in apoptosis, measured by annexin V, in both UM cell lines (Fig. 2D, *p* ≤ 0.0001 for the two highest ASO 3 and highest ASO 11 concentration in 92.1 and *p* < 0.05 for all ASO 3 concentrations and the four highest ASO 11 concentrations in OMM1, one-way ANOVA).

To verify that the observed effects were not related to the lipid-based delivery of the ASOs, we evaluated an alternative non-lipid-based delivery method using the TransIT-X2 transfection reagent. Transfection of 100 nM of ASO 3 significantly decreased *SAMMSON* expression in 92.1 and OMM1 (Supplemental Fig. 4A, *p* < 0.0001, one-way ANOVA). Consequently, *SAMMSON* knockdown was associated with a significant decrease in cell viability in 92.1 and OMM1 (Supplemental Fig. 4B, *p* < 0.0001, one-way ANOVA). Taken together, these results reveal an important role for *SAMMSON* in maintaining UM and CM cell survival in vitro. Due to the genetic similarity between CM and SKCM, where *SAMMSON* has been extensively studied [13, 14], this research will further mainly focus on the role of *SAMMSON* in UM.

### *SAMMSON* inhibition affects protein synthesis and mitochondrial function in UM

**Fig. 3:**
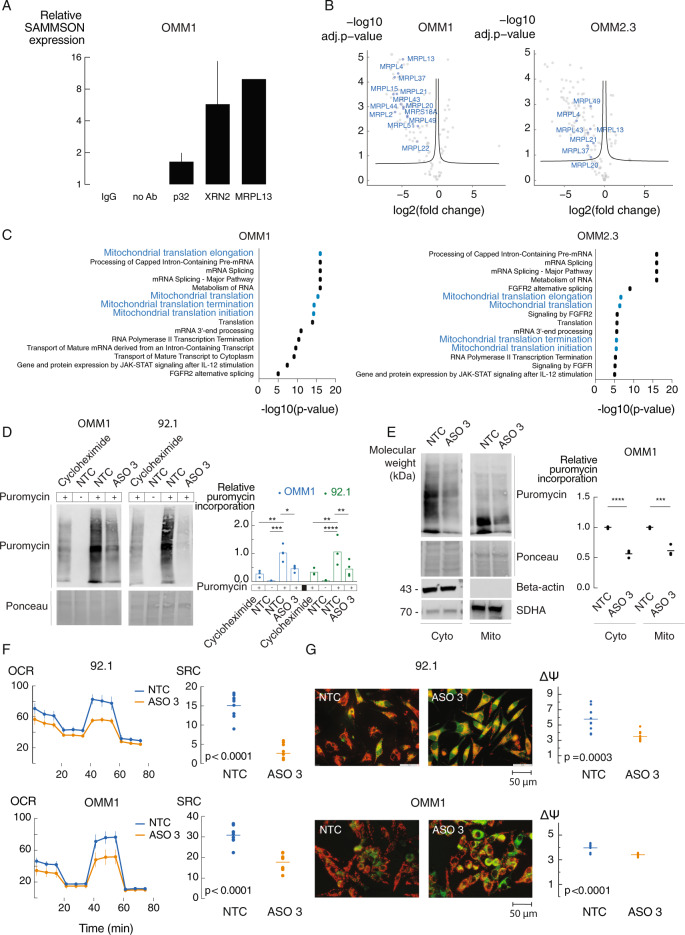
*SAMMSON* inhibition affects translation and mitochondrial function. **A** P32, XRN2 and MRPL13 were identified as *SAMMSON* interacting proteins by means of RIP-qPCR in OMM1. Data presented as the mean ± standard error (SE) of qPCR replicates. **B** Volcano plot depicting *SAMMSON* interacting proteins identified by ChIRP-MS (left). Probes targeting LacZ were included as a control (right). Mitochondrial ribosomal proteins (MRPs) are indicated on the graph. Significance was calculated using two-sided *t*-test. **C** Pathway enrichment analysis for ChIRP results showed participation of *SAMMSON* in mitochondrial translation pathways. **D** Representative images of WB-SUnSET analysis of UM cells treated with cycloheximide (translation inhibitor, positive control), scrambled ASO (NTC) (without puromycin, negative control), NTC ASO or ASO 3 (50 nM). Quantification of protein synthesis measured by calculating the intensity of the puromycin signal on WB. The individual data points (*n* = 3 biological replicates) and mean are presented. *P* values were calculated using two-way ANOVA with Tukey’s multiple comparisons test. **E** Representative images of WB-SUnSET analysis of UM cells treated with scrambled ASO (NTC) or ASO 3 (100 nM) followed by mitochondrial (mito) and cytosolic (cyto) fractionation. Quantification as described in D. The individual data points (*n* = 3 biological replicates) and mean are presented. *P* values were calculated using one-way ANOVA with Tukey’s multiple comparisons test. **F** Oxygen Consumption Rate (OCR) measurements over time after sequential injections of oligomycin, fluoro-carbonyl cyanide phenylhydrazone (FCCP) and rotenone/antimycin A in UM cell lines treated for 24 h with NTC ASO or ASO 3 (100 nM). Data are represented as the mean of three replicates ± s.d. Spare respiratory capacity (SRC) was obtained by subtracting the basal respiration from the maximal respiration. The individual data points and mean are presented. *P* values were calculated using unpaired two-tailed *t*-test. **G** 5,5’,6,6’-Tetraethylbenzimidazolyl-carbocyanine iodide (JC-1) staining in UM cells treated with NTC ASO or ASO 3 (100 nM) (magnification of the images x400). Quantification of the electric membrane potential (ΔΨ) as the red over green fluorescence of 10 random selected fields. The individual data points and mean are presented. *P* values were calculated using unpaired two-tailed *t*-test. *p ≤ 0.05, **p ≤ 0.01, ***p ≤ 0.001, ****p ≤ 0.0001.

To investigate the mechanism by which *SAMMSON* contributes to UM tumor cell survival, we studied *SAMMSON* interaction partners in UM cell lines. First, we evaluated *SAMMSON* binding to p32 and XRN2, 2 interaction partners previously identified in skin melanoma [13, 14], using RIP-qPCR. Immunoprecipitation of both p32 and XRN2 in 92.1 and OMM1 cells revealed a 2- and 6-fold enrichment in OMM1 and a 4- and 64-fold enrichment in 92.1 of *SAMMSON* RNA, respectively, indicating that *SAMMSON* binding to these factors is conserved in uveal melanoma cells (Fig. 3A and Supplemental Fig. 5A). We then applied chromatin isolation by RNA purification and mass spectrometry (ChIRP-MS) in two UM cell lines (OMM1 and OMM2.3) to identify additional interaction partners of *SAMMSON* in UM cells. We verified enrichment of *SAMMSON* RNA upon *SAMMSON* pull down with biotinylated probes (Supplemental Fig. 5B) and subsequently applied mass spectrometry to quantify protein abundance in both the *SAMMSON* and lacZ pull-down samples. Of note, the non-*SAMMSON* binding control LacZ did not result in *SAMMSON* RNA enrichment (data not shown). We detected 83 and 84 proteins that were significantly enriched upon *SAMMSON* pull down in OMM1 and OMM2.3, respectively, of which 57 were found in both cell lines (Fig. 3B, Supplemental Fig. 5C and Supplemental Table 1). We next performed pathway enrichment analysis and found that pathways involved in mitochondrial translation were highly enriched among the candidate interaction partners in both cell lines (Fig. 3C and Supplemental Table 1). Closer inspection of the candidate interaction partners revealed 13 (OMM1) and 7 (OMM2.3) mitochondrial ribosomal proteins (MRPs) (Fig. 3B) that are all part of the 39 S large mitoribosomal subunit involved in mitochondrial translation. One of the identified interaction partners, Mitochondrial Ribosomal Protein L13 (MRPL13), was validated in OMM1 cells by means of immunoprecipitation with a 10-fold enrichment of *SAMMSON* RNA (Fig. 3A). The importance of three MRPs (MRPL4, MRPL13 and MRPL37, all identified as *SAMMSON* interaction partners in both UM cell lines), in maintaining mitochondrial function was assessed by measuring the oxygen consumption rate (OCR) as a proxy for mitochondrial respiration. Differences in OCR after injections of oligomycin, fluoro-carbonyl cyanide phenylhydrazone (FCCP) and rotenone/antimycin A were measured [17]. Knockdown of those MRPs (Supplemental Fig. 5D, *p* < 0.001 for all tested MRP siPOOLs in 92.1 and OMM1, unpaired *t*-test) resulted in a significant decrease in mitochondrial spare respiratory capacity (SRC) of 49% in 92.1 and 32% in OMM1 (Supplemental Fig. 5E, *p* = 0.0008 (92.1) and *p* = 0.0063 (OMM1), unpaired *t*-test). Together with validated p32 and XRN2 interactions, these results suggest a role for *SAMMSON* in regulating translation and mitochondrial function in uveal melanoma. To further investigate the effect of *SAMMSON* knockdown on translation, we quantified global, mitochondrial and cytosolic translation levels using a puromycin incorporation assay (SUnSET [18]). *SAMMSON* knockdown significantly impaired global translation rates by 56% and 61% in UM cell lines OMM1 and 92.1, respectively (Fig. 3D and Supplemental Fig 6A, *p* = 0.0155 (OMM1) and *p* = 0.0077 (92.1), two-way ANOVA). Fractionation revealed a reduction of both mitochondrial and cytosolic translation rates by 38% and 44%, respectively in OMM1 (Fig. 3E and Supplemental Fig 6B, *p* = 0.0002 (mito) and *p* < 0.0001 (cyto), one-way ANOVA).

We then explored the importance of *SAMMSON* for proper mitochondrial function by measuring the OCR. Upon *SAMMSON* knockdown, we observed a significant decrease in mitochondrial spare respiratory capacity (SRC) of 82%, 42% and 72% in 92.1, OMM1 and OMM2.3, respectively (Fig. 3F and Supplemental Fig 7A, *p* < 0.0001, unpaired *t*-test). This was further verified by a 5,5′,6,6′-tetraethylbenzimidazolyl-carbocyanine iodide (JC-1) fluorescence staining, where the electric membrane potential (ΔΨ) is quantified based on the relative red over green fluorescence [19]. *SAMMSON* knockdown decreases ΔΨ with 39%, 14% and 31% in 92.1, OMM1 and OMM2.3, respectively, indicating oxidative phosphorylation (OXPHOS) impairment (Fig. 3G and Supplemental Fig 7B, *p* = 0.0003 (92.1) and *p* < 0.0001 (OMM1 and OMM2.3), unpaired *t*-test). These data suggest that the mechanism by which *SAMMSON* affects translation is conserved between UM and SKCM, resulting in an impairment of mitochondrial function. In addition, knockdown of MRPL13, together with Mitochondrial Ribosomal Protein L4 and L37 (MRPL4 and MRPL37), three *SAMMSON* interaction partners identified in both OMM1 and OMM2.3, resulted in a significant decrease in spare respiratory capacity of 49% in 92.1 and 32% in OMM1, demonstrating their importance in mitochondrial function.

To assess the importance of mitochondrial translation in UM cell survival, *SAMMSON* expressing UM cell lines 92.1 and OMM1 and non-*SAMMSON* expressing cell lines CT5.3hTERT and HEK293T were treated with tigecycline, a bacteriostatic antibiotic of the tetracyclines family [20–23]. In both UM cell lines, we observed a tigecycline dose-dependent reduction in cell confluence and induction of apoptosis (Supplemental Fig 8A, *p* < 0.0001 (confluence) and *p* < 0.0001 for all concentrations, except 3.125 µM in 92.1 and p≤0.01 for the two highest concentrations in OMM1 (apoptosis), one-way ANOVA), verifying that UM cells strongly depend on mitochondrial translation. While the growth of non-*SAMMSON* expressing cells HEK293T and CT5.3hTERT is also affected by tigecycline treatment (Supplemental Fig 8B, *p* < 0.0001 (HEK293T) and *p* ≤ 0.05 for all concentrations, except 3.125 µM (CT5.3hTERT), two-way ANOVA), tigecycline was markedly less effective in CT5.3hTERT cells (cell viability reduction of 35% using 50 µM of tigecycline compared to ≥55% in 92.1, OMM1 and HEK293T).

**Fig. 4:**
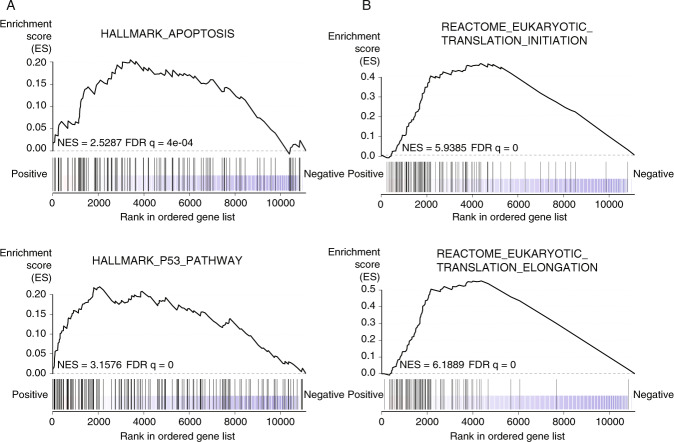
Enrichment of apoptosis, p53 and translation gene sets upon *SAMMSON* knockdown. **A**, **B** GSEA results for Hallmark gene sets apoptosis and p53 (MsigDB) (**A**) and c2 curated gene sets translation initiation and translation elongation (MsigDB) (**B**) that are enriched upon ASO 3 treatment (50 nM). Normalized enrichment score (NES) and false discovery rate (FDR) are depicted on the enrichment plots.

To assess the impact of *SAMMSON* on the UM transcriptome, we performed shallow RNA sequencing of UM cell lines 92.1 and OMM1 treated with either NTC ASO or ASO 3. Differential gene expression analysis revealed 378 up- and 255 downregulated genes (adjusted *p* value <0.05). Gene set enrichment analysis (GSEA) revealed a significant upregulation of gene sets associated with apoptosis and p53 response upon *SAMMSON* silencing (Fig. 4A, FDR *q* = 4e–04 (apoptosis) and FDR *q* = 0 (p53) and Supplemental Table 2), supporting our phenotypic observations. Surprisingly, *SAMMSON* inhibition also resulted in a transcriptional activation of genes involved in translation (Fig. 4B, FDR *q* = 0).

### *SAMMSON* inhibition suppresses UM PDX growth

**Fig. 5:**
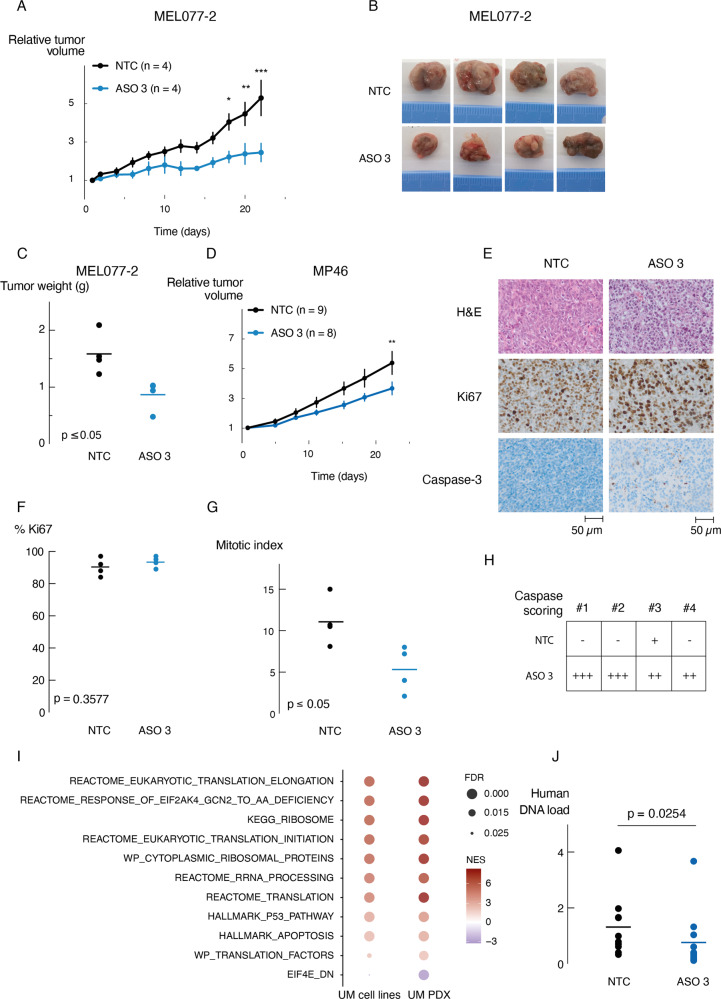
*SAMMSON* inhibition reduces tumor growth in vivo. **A** Relative tumor volume of MEL077 PDX mice (MEL077-2) subcutaneously injected with NTC ASO or ASO 3 (10 mg/kg). Data are mean ± s.e.m. of multiple replicates (*n* = 4). *P* values were calculated using two-way ANOVA with Sidak’s multiple testing correction. **B** Representative tumors of MEL077 PDX mice (MEL077-2) 22 days after treatment (n = 4/treatment group). **C** Tumor weight of tumors as shown in B. Individual data points and mean are presented. *P* value was calculated using unpaired two-tailed *t*-test. **D** Relative tumor volume of MP46 PDX mice subcutaneously injected with scrambled ASO (NTC) or ASO 3 (10 mg/kg). Data are mean ± s.e.m. of multiple replicates (*n* = 8–9/treatment group). *P* values were calculated using two-way ANOVA with Sidak’s multiple testing correction. **E** Representative images of H&E, Ki67 and Caspase-3 staining of MEL077 tumor sections (MEL077-2) 22 days after treatment with NTC ASO or ASO 3. **F** Percentage of Ki67 positive cells in 500 counted cells per tumor sample (MEL077-2, *n* = 4/treatment group) (magnification x100). Individual data points and mean are presented. *P* value was calculated using unpaired two-tailed *t*-test. **G** Number of mitotic cells based on H&E staining (MEL077-2) in E of 10 random high power fields (HPFs) (magnification x400). Each data point represents the mean of 10 HPFs per tumor sample (*n* = 4/treatment group). *P* value was calculated using unpaired two-tailed *t*-test. **H** Caspase-3 scoring based on morphology and staining intensity as shown in E of four tumor samples per treatment group (MEL077-2). **I** Selected GSEA results of RNA seq data from UM cell lines OMM1 and 92.1 and UM MEL077 PDX tumors (MEL077-2) demonstrating overlapping enrichment of gene sets involved in apoptosis, p53 and translation upon ASO 3 treatment. The depth of the color represents the normalized enrichment score (NES). The area of the circle represents the false discovery rate (FDR). **J** Human DNA load measured in lung tissues of MEL077-2 (*n* = 4/treatment group) and MP46 (*n* = 10–11/treatment group) mice by means of qPCR of Alu-Sq repetitive sequence. Individual data points and mean are presented. *P* value was calculated using unpaired one-tailed *t*-test. *p ≤ 0.05, **p ≤ 0.01, ***p ≤ 0.001.

In vivo anti-tumor effects of *SAMMSON* knockdown were evaluated in three independent experiments using two UM patient derived xenografts (PDX) models (MP46 (GNAQ^Q209L^) and MEL077 (derived from a patient progressing on the immune checkpoint inhibitor pembrolizumab)) showing high *SAMMSON* expression levels (Fig. 1D). When the tumor xenografts reached a volume of 60–180 mm^3^, the mice were randomly separated into two groups and were subcutaneously injected with either NTC ASO or ASO 3 (10 mg/kg). Tumor growth, monitored for 22 days, was significantly delayed (MEL077-2, Fig. 5A, *p* = 0.0001 at endpoint, two-way ANOVA) and tumor weight significantly reduced in MEL077 PDX mice treated with ASO 3 compared to NTC ASO (MEL077-2, Fig. 5B, C, *p* = 0.0183, unpaired *t*-test). A similar trend could be observed in an independent repeat in PDX model MEL077 (MEL077-1, Supplemental Fig 9A) and a significant delay in tumor growth was also observed in UM PDX model MP46 (Fig. 5D, p = 0.0050 at endpoint, two-way ANOVA). Notably, the mice of both PDX models did not suffer from any weight loss after ASO 3 treatment (Supplemental Fig 9B-D).

Immunohistochemistry (IHC) analysis with anti-Ki67 of the MEL077 model revealed highly aggressive tumors, with over 80% of proliferating cells in all tumor samples (Fig. 5E). While the difference in Ki67 expression between the ASO 3 and NTC ASO treated mice was too small to be statistically significant (Fig. 5E, F, *p* = 0.3577, unpaired t-test), further investigation of the mitotic index showed a significant reduction in the number of mitotic cells in the ASO 3 treated tumor samples (Fig. 5E, G, *p* = 0.0278, unpaired t-test). The discrepancy between Ki67 and MI results might be explained by the selected regions and cell cycle phase coverage of both methods, which has also been described in other tumors [24–26]. In addition, caspase-3 activity was elevated in tumors from mice treated with ASO 3 compared to NTC ASO, indicating *SAMMSON* knockdown induces apoptosis in vivo (Fig. 5E, H). Moreover, all tumor samples were correctly labeled as ‘ASO 3-treated’ or ‘NTC ASO-treated’, independently by two pathologists and blinded to treatment.

To further investigate these tumors at the molecular level, we applied RNA-sequencing to generate transcriptome profiles to define pathways significantly enriched or repressed upon PDX ASO treatment. Notably, we observed a strong overlap (*p* < 0.0001, Fisher exact test) between pathways induced in the PDX model and pathways induced in the UM cell lines upon *SAMMSON* knockdown. Gene sets related to apoptosis and p53 response were significantly upregulated (Fig. 5I, FDR *q* < 0.05, Supplemental Table 2). Similarly, pathways related to translation were also found transcriptionally upregulated. Median *SAMMSON* expression was decreased in tumors from ASO 3 treated mice however this difference was not significant (Supplemental Figure 9E).

Although no macro-metastatic lesions were observed in both PDX models, we investigated the presence of tumoral DNA in blood and various murine tissues resected from both PDX models [27]. To this end, we extracted genomic DNA (gDNA) from blood, liver and lung of PDX mice that received ASO 3 or NTC ASO treatment and performed quantitative PCR using the human specific Alu-Sq, SVA and LINE-1 repetitive sequences as an indicator of tumor load in these tissues. Copy number levels of the murine Hprt1 and Pthlh genes were used for normalization. Tumor DNA was only detected in the murine lung tissue samples and compared to NTC ASO treated mice, mice treated with ASO 3 had significantly reduced levels of tumor DNA in lung tissue (Fig. 5J and Supplemental Fig 9F, G, *p* = 0.0254 (Alu-Sq), *p* = 0.0357 (LINE-1), *p* = 0.0202 (SVA), unpaired t-test).

## Discussion

Long non-coding RNAs are crucial players in many cellular and biological processes including regulation of gene expression, cell growth, differentiation and development. Aberrant expression of lncRNAs is observed in virtually all tumor types and some lncRNAs appear to be cell type- and tissue-specific [28, 29]. The lncRNA *SAMMSON* was discovered as a melanoma specific lncRNA [13], but several studies have also shown occasional expression in gastric cancer (GC), hepatocellular carcinoma (HCC), glioblastoma (GBM) and papillary thyroid carcinoma (PTC) [30–34]. In the present study, we found that *SAMMSON* is expressed in more than 80% of uveal melanoma tumors at levels that are substantially higher compared to non-melanoma tumors. Furthermore, *SAMMSON* is significantly higher expressed in metastatic UM tumors compared to matching primary tumors. In addition, *SAMMSON* expression could be observed in conjunctival melanoma (CM) cells that are genetically and phenotypically more related to skin melanoma. While 50% of uveal melanoma tumors are characterized by loss of an entire copy of chromosome 3, *SAMMSON* expression levels are not reduced in monosomy 3 tumors whereas the majority of genes on chromosome 3 do show a clear gene-dosage effect. These observations suggest the existence of a compensation mechanism that requires further investigation. Potentially, monosomy 3 tumors upregulate one or multiple transcription factors that drive *SAMMSON* expression from the remaining allele.

The lack of treatments for UM patients with metastatic dissemination that occurs in 50% of the patients and is accompanied by extremely poor survival [4] demonstrates the high unmet need for new treatment modalities. Therapeutic nucleic acid-based approaches like siRNA and antisense oligonucleotides (ASO) hold enormous potential to target RNA molecules, including lncRNAs. Advances in therapeutic ASO and siRNA technology research, such as the development of locked nucleid acid (LNA) and S-constrained ethyl (cEt) modified ASOs and 2’-OMe and 2’-fluoro modified siRNAs, enabled the development and approval of several ASO and siRNA drugs to treat diseases such as spinal muscular atrophy (SMA) and hereditary transthyretin-mediated amyloidosis (hATTR) [35–37]. Our work demonstrates that, similar to skin melanoma, ASO-mediated *SAMMSON* knockdown in various in vitro and in vivo UM models induces a potent anti-tumor response including growth reduction, induction of apoptosis, and reduced levels of tumor-derived DNA in lung tissue. Whether this DNA is cell-free or derived from tumor cells invading these tissues remains to be investigated. The fact that we did not observe tumor DNA in blood may be indicative of the latter. Additional studies in uveal melanoma xenograft models that metastasize [38–40] should further assess the impact of *SAMMSON* knockdown on metastatic disease. Furthermore, the in vivo kinetics of *SAMMSON* expression upon ASO treatment should be explored in more detail. While we observed a trend of *SAMMSON* knockdown in vivo 3 weeks after start of treatment, the difference was not significant. Differences in baseline *SAMMSON* expression levels and (time-dependent) variability in knockdown efficiency between mice may explain these observations. *SAMMSON* expression quantification at earlier treatment time points could provide more insights in knockdown dynamics. Our in vitro data, based on multiple cell lines with a different genetic background, and in vivo data, based on two genetically different UM PDX models, suggest that the genetic background of the tumor does not influence the response to *SAMMSON* inhibition. Furthermore, cell lines originating from primary and metastatic UM tumors showed similar responses to *SAMMSON* knockdown, including viability reduction and induction of apoptosis. Since *SAMMSON* expression levels are elevated in metastatic tumors, these results highlight the broad relevance of *SAMMSON* inhibition as a potential therapeutic strategy, which may be primarily of interest for metastatic UM patients, where the therapeutic need is high. On-target toxicity of the ASO treatment could not be investigated due to the absence of a *SAMMSON* homologue in the mouse genome, which is a limitation of the current study. On the other hand, the fact that *SAMMSON* is expressed in most uveal melanoma tumors (irrespective of genetic background or tumor stage) and absent in differentiated human tissues [13] suggests that therapeutic targeting of *SAMMSON* could selectively kill tumor cells without on-target toxicity in other tissues.

Earlier studies in SKCM demonstrated *SAMMSON* interaction with proteins involved in mitochondrial and cytosolic translation, such as p32, XRN2 and CARF [14]. In UM, interactions with p32 and XRN2 were confirmed and novel candidate interactions with multiple proteins belonging to the 39S large mitoribosomal subunit were identified, of which interaction with MRPL13 was validated, further supporting the role of *SAMMSON* in regulating mitochondrial and cytosolic translation. Additional mechanistic studies are required to further elucidate the role of these novel candidate interactors. Of note, XRN2 and p32 were not identified by mass-spectrometry analysis, suggesting that we may have missed other interaction partners. In support of these interactions and the established role of *SAMMSON* in SKCM, *SAMMSON* inhibition effectively impairs mitochondrial and cytosolic translation rates. Since all proteins required for mitochondrial translation, including the mitoribosomal proteins and the mitochondrial translation factors, are translated by cytosolic ribosomes [41], further investigation is needed to determine whether the observed inhibition on mitochondrial translation is a direct or indirect effect. Furthermore, all 13 polypeptides synthesized by the mitochondrial ribosomes (mitoribosomes) are essential components of the oxidative phosphorylation machinery [42]. *SAMMSON* inhibition indeed impairs mitochondrial function, as shown in three UM cell lines. Moreover, knockdown of the identified *SAMMSON* interaction partners MRPL4, MRPL13 and MRPL37 also impaired mitochondrial respiration, demonstrating their involvement in mitochondrial function. This results in mitochondrial precursor overaccumulation stress (mPOS), characterized by the toxic accumulation and aggregation of unimported mitochondrial proteins in the cytosol [43]. Cells need to balance between import of mitochondrial proteins and the cytosolic capacity to handle unimported mitochondrial proteins. Induction of mPOS can tip that balance, hereby compromising cell viability. Although *SAMMSON* inhibition affects global translation, transcriptome analysis in both UM cell lines and PDX models revealed a transcriptional upregulation of various components involved in the translational machinery. These observations have also been observed in yeast when using translation inhibitors such as cycloheximide (CHX) [44] and upregulation in ribosomal biogenesis (RiBi) gene expression has been observed when inducing ribosomal stress which lowers the translational capacity [45]. Ribosomal biogenesis is one of the most energy consuming processes, which requires a coordinated de-repression and active induction of RiBi gene expression [46]. Transcriptional upregulation of these genes while global translation is affected, suggest the existence of a feedback loop to compensate for the loss in translational capacity.

In conclusion, our work shows that inhibition of *SAMMSON* impairs cytosolic and mitochondrial translation, which consequently affects mitochondrial function and ultimately results in decreased cell viability and induction of apoptosis, both in vitro and in vivo. Together, our findings suggest that *SAMMSON* is an attractive therapeutic target for UM and that *SAMMSON* can be targeted in vivo using ASO technology.

## Material and methods

### Cell culture

For all human cell lines, an ethical approval was obtained from the Ghent University commission for medical ethics. The human uveal melanoma cell lines 92.1 [47], OMM1 [16], OMM2.3, MEL270, MP38 [48], MP46 [48], MM28 [48] and MP65 [48] and conjunctival melanoma cell lines CRMM1 and CRMM2 [49] were obtained from the Leiden University Medical Center, The Netherlands. OMM2.3 and MEL270 were a kind gift from Bruce Ksander. MM28, MP65, MP46 and MP38 were a kind gift from Sergio Roman-Roman. Uveal melanoma cell line MEL077 and skin melanoma cell line SK-MEL28 was obtained from the Laboratory for Molecular Cancer Biology, VIB-KU Leuven, Belgium. *SAMMSON* negative cell lines HEK293T and CT5.3hTERT were obtained from ATCC (Manassas, Virginia, USA) and the Department of Radiation oncology and experimental cancer research, Ghent University, Belgium, respectively. The cell lines 92.1, OMM1, OMM2.3 and MEL270 were grown in Dulbecco’s modified Eagle’s medium (DMEM, Gibco)/F12––Roswell Park Memorial Institute (RPMI, Gibco) 1640 (1:1) medium. SK-MEL28 was grown in DMEM/F-12 GlutaMAX medium (Gibco), CT5.3hTERT in DMEM medium, CRMM1 and CRMM2 in Ham’s F-12K (Kaighn’s) medium (Gibco), MM28, MP65, MP46 and MP38 in Iscove’s Modified Dulbecco’s Medium (IMDM) and HEK293T and MEL077 in RPMI 1640 medium. Media of MM28, MP65, MP46 and MP38 were supplemented with 20% fetal bovine serum (FBS) and all other media with 10% FBS, 2 mM L-glutamine (Gibco) and 100 IU/ml penicillin/streptomycin (Gibco) and all cell lines were incubated in a humidified atmosphere containing 5% CO_2_ at 37 °C. For executing experiments, cells were grown in L-glutamine and penicillin/streptomycin free media. Short tandem repeat (STR) genotyping was used to validate cell line authenticity and absence of mycoplasma was verified on a monthly basis for all cell lines in culture.

## Supplementary information

Supplementary legends

Supplementary methods

Supplemental Fig 1. *SAMMSON* expression is independent from patient survival, tumor stage, tumor localization site and metastatic state of the UM patient.

Supplemental Fig 2. Expressed genes in monosomy 3 UM tumors compared to disomy 3 UM tumors.

Supplemental Fig 3. *SAMMSON* knockdown affects cell growth in CM cell line CRMM1.

Supplemental Fig 4. Non-lipid-based delivery of ASOs in UM cells.

Supplemental Fig 5. Identification of *SAMMSON* interaction partners.

Supplemental Fig 6. Uncropped images of WB-SUnSET analysis.

Supplemental Fig 7. Impairment of mitochondrial function upon *SAMMSON* knockdown in UM cell line OMM2.3.

Supplemental Fig 8. Phenotypic results using tigecycline are comparable to *SAMMSON* inhibition in UM cells.

Supplemental Fig 9. *SAMMSON* inhibition slows down tumor growth in vivo.

Supplemental Table 1.

Supplemental Table 2.
