# Correction to: The long non-coding RNA *SAMMSON* is essential for uveal melanoma cell survival

**DOI:** 10.1038/s41388-021-02051-6

**Published:** 2021-11-15

**Authors:** Shanna Dewaele, Louis Delhaye, Boel De Paepe, Eric James de Bony, Jilke De Wilde, Katrien Vanderheyden, Jasper Anckaert, Nurten Yigit, Justine Nuytens, Eveline Vanden Eynde, Joél Smet, Maxime Verschoore, Fariba Nemati, Didier Decaudin, Manuel Rodrigues, Peihua Zhao, Aart Jochemsen, Eleonora Leucci, Jo Vandesompele, Jo Van Dorpe, Jean-Christophe Marine, Rudy Van Coster, Sven Eyckerman, Pieter Mestdagh

**Affiliations:** 1 OncoRNALab, Center for Medical Genetics (CMGG), Ghent University, Ghent, Belgium; 2 Cancer Research Institute Ghent (CRIG), Ghent, Belgium; 3 Department of Biomolecular Medicine, Ghent University, Ghent, Belgium; 4 Center for Medical Biotechnology, VIB-Ghent University, Ghent, Belgium; 5 Department of Pediatrics, Division of Pediatric Neurology and Metabolism, Ghent University Hospital, Ghent, Belgium; 6 Department of pathology, Ghent University Hospital, Ghent, Belgium; 7 Institut Curie, Laboratory of Preclinical Investigation, Translational Research Department, PSL Research University, Paris, France; 8 Institut Curie, Department of Medical Oncology, PSL Research University, Paris, France; 9 Inserm U830, DNA Repair and Uveal Melanoma (D.R.U.M.), Equipe labellisée par la Ligue Nationale Contre le Cancer, Institut Curie, PSL Research University, Paris, 75005 France; 10 Center for Medical Biotechnology, VIB-KU Leuven Center for Cancer Biology, Leuven, Belgium; 11 Laboratory for RNA Cancer Biology, Department of Oncology, KU Leuven, Leuven, Belgium; 12 Department of Cell and Chemical Biology, Leiden University Medical Center, Leiden, The Netherlands; 13 TRACE, LKI Leuven Cancer Institute, Leuven, Belgium; 14 Laboratory for Molecular Cancer Biology, Center for Cancer Biology, VIB, Leuven, Belgium; 15 Laboratory for Molecular Cancer Biology, Department of Oncology, KU Leuven, Leuven, Belgium

**Keywords:** Targeted therapies, Non-coding RNAs

Correction to: *Oncogene* 10.1038/s41388-021-02006-x, published online 10 September 2021

In this article the affiliation details for Author Jean-Christophe Marine were incorrectly given as Laboratory for RNA Cancer Biology, Department of Oncology, KU Leuven, Leuven, Belgium. but should have been Laboratory for Molecular Cancer Biology, Center for Cancer Biology, VIB, Leuven, Belgium; Laboratory for Molecular Cancer Biology, Department of Oncology, KU Leuven, Leuven, Belgium.

The original article has been corrected.

