# Supplementary material for: The long non-coding RNA SAMMSON is essential for uveal melanoma cell survival
Source: Oncogene. 2021 Sep 10;41(1):15–25. doi: 10.1038/s41388-021-02006-x (PMC8724009; doi:10.1038/s41388-021-02006-x)
Supplement: Supplementary file 1 — Supplementary legends [file 41388_2021_2006_MOESM1_ESM.docx]

**Supplemental Fig 1. *SAMMSON* expression is independent from patient survival, tumor stage, tumor localization site and metastatic state of the UM patient.** RNA sequencing data from >10 000 tumor samples and 32 cancer types (TCGA) showing no correlation between *SAMMSON* expression and the vital status, clinical stage, primary tumor site or pathological metastatic state of the UM patient. n.s. = not significant.

**Supplemental Fig 2. Expressed genes in monosomy 3 UM tumors compared to disomy 3 UM tumors.** 656 genes are expressed in monosomy 3 tumors of which 581 (89%) are downregulated and 75 (11%) upregulated compared to disomy 3 tumors (p<0.001, Mann-Whitney test).

**Supplemental Fig 3. *SAMMSON* knockdown affects cell growth in CM cell line CRMM1. A.** Relative *SAMMSON* expression of CM cell line CRMM1 48h after transfection with 5, 10, 25, 50 or 100 nM of NTC ASO, ASO 3 or ASO 11 and scaled to untreated cells (Mock). The individual data points and mean of NTC ASO, ASO 3 and ASO11 are presented. The mock treated data are presented as the mean of 4 replicates ± s.d. P-values were calculated for each ASO 3/ASO 11 concentration and compared to its corresponding NTC ASO concentration using one-way ANOVA with Dunnett’s multiple testing correction. **B**. Reduction of proliferation in CRMM1 upon transfection with 5, 10, 25, 50 or 100 nM ASO 3 or ASO 11 compared to NTC ASO treatment or untreated cells (mock) measured by time-lapse microscopy (every 2-3 h) using the IncuCyte device. Data represents mean ± s.d. of 3 replicates. NTC ASO data represents the mean ± s.d. of all NTC ASO concentrations (5, 10, 25, 50 and 100 nM). P-values were calculated at the 72 h time point compared to all NTC ASO concentrations using one-way ANOVA with Dunnett’s multiple testing correction. ** p≤0.01, *** p≤0.001, **** p≤0.0001.

**Supplemental Fig 4. Non-lipid-based delivery of ASOs in UM cells. A**. Relative *SAMMSON* expression in two UM cell lines transfected with scrambled ASO (NTC ASO) or ASO 3 (100 nM) using the TransIT-X2 transfection reagent. The results are obtained 48 h after transfection and the data points of six replicates and the mean are presented. P-values were calculated using one-way ANOVA with Tukey’s multiple comparisons test. **B** Viability (Cell titer glo) assay in NTC ASO and ASO 3 treated UM cells using the TransIT-X2 transfection reagent (72 h post-transfection). The data are presented as the mean of 10 replicates ± s.d. P-values were calculated using one-way ANOVA with Tukey’s multiple comparisons test. **** p≤0.0001.

**Supplemental Fig 5. Identification of *SAMMSON* interaction partners. A.** p32 and XRN2 were identified as *SAMMSON* interacting proteins in UM cell line 92.1 by means of RIP-qPCR. **B.** Enrichment of *SAMMSON* RNA upon *SAMMSON* pull down with biotinylated probes in ChIRP-MS. Data represents mean of 3 replicates ± s.d. LacZ pull down, included as a control, did not result in *SAMMSON* RNA enrichment (data not shown). **C**. Significantly enriched proteins upon *SAMMSON* pull down in two UM cell lines with 57 overlapping proteins. **D.** Relative *MRPL4, MRPL13* or *MRPL37* expression in UM cell lines 92.1 and OMM1 48h after transfection with 100 nM of scrambled (NTC) siPOOLs, MRPL4, MRPL13 or MRPL37 siPOOLs. The 3 individual data points and mean are presented. P-values were calculated using an unpaired two-tailed t-test. **E.** Oxygen Consumption Rate (OCR) measurements over time after sequential injections of oligomycin, fluoro-carbonyl cyanide phenylhydrazone (FCCP) and rotenone/antimycin A in UM cell lines treated for 72 h with NTC siPOOLs or MRPL4, MRPL13 and MRPL37 siPOOLs (100 nM). Data are represented as the mean of 3 replicates ± s.d. Spare respiratory capacity (SRC) was obtained by subtracting the basal respiration from the maximal respiration. The individual data points and mean are presented. P-values were calculated using unpaired two-tailed t-test. ** p≤0.01, *** p≤0.001, **** p≤0.0001.

**Supplemental Fig 6. Uncropped images of WB-SUnSET analysis. A**. Uncropped images of WB-SUnSET analysis of UM cells treated with cycloheximide (translation inhibitor, positive control), scrambled ASO (NTC) (without puromycin, negative control), NTC ASO or ASO 3 (50 nM). **B**. Uncropped images of WB-SUnSET analysis of UM cells treated with scrambled ASO (NTC) or ASO 3 (100 nM) followed by mitochondrial (mito) and cytosolic (cyto) fractionation.

**Supplemental Fig 7. Impairment of mitochondrial function upon *SAMMSON* knockdown in UM cell line OMM2.3. A**. Oxygen Consumption Rate (OCR) measurements over time after sequential injections of oligomycin, fluoro-carbonyl cyanide phenylhydrazone (FCCP) and rotenone/antimycin A in UM cell line OMM2.3 treated for 24 h with NTC ASO or ASO 3 (100 nM). Data are represented as the mean of 3 replicates ± s.d. Spare respiratory capacity (SRC) was obtained by subtracting the basal respiration from the maximal respiration. The individual data points and mean are presented. P-values were calculated using unpaired two-tailed t-test. **B**. 5,5’,6,6’-Tetraethylbenzimidazolyl-carbocyanine iodide (JC-1) staining in UM cell line OMM2.3 treated with NTC ASO or ASO 3 (100 nM) (magnification of the images x400). Quantification of the electric membrane potential (ΔΨ) as the red over green fluorescence of 10 random selected fields. The individual data points and mean are presented. P-values were calculated using unpaired two-tailed t-test.

**Supplemental Fig 8. Phenotypic results using tigecycline are comparable to *SAMMSON* inhibition in UM cells. A**. Reduction in proliferation (confluence) and induction of apoptosis (annexin V) in UM cell lines 92.1 and OMM1 upon treatment with multiple concentrations of tigecycline (3.125, 6.25, 12.5, 25 or 50 µM) compared to untreated cells (0 µM) measured with time-lapse microscopy (every 2-3 h) using the IncuCyte device. **B**. Relative confluence 48 h after treatment with multiple concentrations of tigecycline (3.125, 6.25, 12.5, 25 or 50 µM) compared to untreated cells (0 µM). Data in A and B represent mean ± s.d. of 5 replicates. P-values are calculated using one-way ANOVA with Dunnett’s multiple comparisons test (last time point for A). * p≤0.05, ** p≤0.01, *** p≤0.001, **** p≤0.0001.

**Supplemental Fig 9. *SAMMSON* inhibition slows down tumor growth *in vivo*. A.** Relative tumor volume of MEL-077 PDX mice (MEL077-1) subcutaneously injected with NTC ASO or ASO 3 (10 mg/kg). Data are mean ± s.e.m. of multiple replicates (n=5/treatment group). **B-D.** Relative weight of MEL-077 PDX mice (B, C) or MP46 PDX mice (D) treated with scrambled ASO (NTC) or ASO 3 (10 mg/kg, 3.5 times/week) for 3 weeks. Data are mean ± s.e.m. of multiple replicates (n=4 (MEL077–2), n=5 (MEL077–1), n=8-9 (MP46) per treatment group). **E.** RNA sequencing data of UM tumors collected from MEL-077 PDX mice (MEL077-2) after 22 days of treatment, which show a downward trend (not significant) in *SAMMSON* expression levels in ASO 3 treated tumors compared to NTC ASO treated tumors (n=4 per treatment group). **F, G.** Human DNA load measured in lung tissues of MEL077 (n=4/ treatment group) and MP46 (n=10-11/treatment group) mice by means of qPCR for LINE-1 (F) or SVA (G) repetitive sequence. P-value was calculated using unpaired one-tailed t-test.

**Supplemental Table 1.** ChIRP-MS data in OMM1 and OMM2.3

**Supplemental Table 2.** GSEA of UM PDX tumors and UM cell lines
