# Supplementary material for: The long non-coding RNA SAMMSON is essential for uveal melanoma cell survival
Source: Oncogene. 2021 Sep 10;41(1):15–25. doi: 10.1038/s41388-021-02006-x (PMC8724009; doi:10.1038/s41388-021-02006-x)
Supplement: Supplementary file 2 — Supplementary methods [file 41388_2021_2006_MOESM2_ESM.docx]

**Supplementary methods**

**Animal models**

The *in vivo* experiment with PDX model MEL077 (ref 1) was executed at the Patient Derived Tumor Xenograft Platform, Trace, Leuven, Belgium. The experimental protocol and animal housing were in accordance with institutional guidelines as put forth by the Belgian Ethical Committee and the KU Leuven animal ethics committee that approved this study (agreement P164/2019, KU Leuven, Belgium). Female Naval Medical Research Institute (NMRI) mice were engrafted interscapular at an age of 12 weeks with an UM tumor fragment of 3 mm^3^. Mice bearing tumors with a volume of 150 mm^3^ were individually identified and blinded and randomly assigned to the different treatment/control groups. Mice were weighed twice a week. Tumor volumes were calculated by measuring every 2 days the length (L), width (W) and height (H) of the tumor with calipers. Each tumor volume (V) was calculated according to the following formula: V = (LxWxH)x(π/6). Tumor volume measurements occurred not blinded. At the end of treatment, mice were sacrificed with collection of tumor, liver, lung and blood. ASO 3 (n=4) and NTC ASO (n=4) were injected subcutaneously on the hip at a dose of 10 mg/kg 3.5 times a week. An independent repeat of this experiment has been performed using an independent mouse cohort (n=5/treatment group).

The *in vivo* experiment with PDX model MP46 (BAP1 negative, GNAQ^mut^, monosomy 3) was executed at the Institut Curie, Paris, France. Animal care and use for this study were performed in accordance with the recommendations of the European Community (2010/63/UE) for the care and use of laboratory animals. Experimental procedures were specifically approved by the ethics committee of the Institut Curie CEEA-IC #118 (Authorization APAFiS# 25870-2020060410487032-v1 given by National Authority) in compliance with the international guidelines. Female severe combined immunodeficiency (SCID) mice were engrafted with an UM tumor fragment of 15 mm^3^. Mice bearing tumors with a volume of 60 - 180 mm^3^ were individually identified and randomly assigned to the control or treatment groups. Mice were weighed and tumors were measured twice a week. Tumor volume measurements occurred not blinded. Tumor volumes were calculated by measuring two perpendicular diameters with calipers. Xenografted mice were sacrificed at the end of the treatment. Each tumor volume (V) was calculated according to the following formula: V = a x b^2^/2, where a and b are the largest and smallest perpendicular tumor diameters. ASO 3 (n=8) and NTC ASO (n=9) were administered subcutaneously in the peritumoral area at a dose of 10 mg/kg 3 times a week in the first week of treatment and twice a week in the following weeks. Relative tumor volumes (RTV) were calculated with the following formula: RTV = (Vx/V1), where Vx and V1 are the tumor volumes on day x and the first day of treatment, respectively. The number of animals used is based on previously published *in vivo* work using a *SAMMSON* inhibiting ASO^2^.

**Compounds, siPOOLs and antisense oligonucleotides (ASOs)**

Tigecycline was purchased from Selleckchem (Bio-Connect, Huissen, The Netherlands).

MRPL4 (GeneID: 51073), MRPL13 (GeneID: 28998) and MRPL37 (GeneID: 51253) siPOOLs were purchased from siTOOLs Biotech (Planegg/Martinsried, Germany).

The LNA GapmeR oligonucleotides specifically targeting *SAMMSON* and the LNA non-targeting control (NTC) GapmeR were purchased from Qiagen (Hilden, Germany, catalog number 339517).

Sequence LNA ASO 3: GTGTGAACTTGGCT

Sequence LNA ASO 11: TTTGAGAGTTGGAGGA

Sequence LNA NTC ASO: TCATACTATATGACAG

**Cell proliferation and apoptosis assay**

Cells were seeded in 96 well plates (Corning costar 3596) at a density of 5000 cells/well and were allowed to settle overnight. Subsequently, the cells were transfected with ASO 3 or NTC ASO using lipofectamine 2000 (Thermo Fisher Scientific, Waltham, Massachusetts, USA) or TransIT-X2 (Mirus Bio, Madison, Wisconsin, USA). For those assays, a minimum of 3 technical replicates per treatment group were included to obtain enough statistical power.

Cell viability and apoptosis were examined using a CellTiter-Glo assay (Promega, Madison, Wisconsin, USA) and Caspase 3/7 assay (Promega), respectively. Before initiating the assay, the culture plates and reconstituted assay buffer were placed at room temperature for 30 minutes. Next, the culture medium was replaced by 200 µl fresh culture medium - assay buffer (1:1) mix. To induce complete cell lysis, the plates were shaken during 10 min. 100 µl from each well was subsequently transferred to an opaque 96-well plate (Nunc), which was measured with a GloMax 96 Microplate Luminometer (Promega).

For real-time analysis, the IncuCyte Zoom system and IncuCyte S3 system (Essen BioScience, Newark, England) were used. Cells were seeded and treated as described above. After treatment, the culture plate was incubated in an IncuCyte Zoom system or IncuCyte S3 system at 37°C in a humidified 5% CO_2_ incubator. Phase contrast and red fluorescent whole well images were captured every 3 h. The IncuCyte ZOOM (version 2016B) and IncuCyte S3 software (version 2019B) (Essen BioScience) were utilized in real-time to measure % confluence, as a proxy for proliferation. The % confluence values were corrected for seeding variability resulting in normalized % confluence values. For apoptosis assessment, annexin V (Essen BioScience, dilution 1:400) was added while treating the cells. The IncuCyte ZOOM and IncuCyte S3 software (Essen BioScience) were utilized in real-time to measure % confluence and total Red object area (µm^2^/well). The ratio between total Red object area and phase object confluence (%) is resulting in relative annexin V ratios.

**SUnSET**

Cells were seeded in T75 culture flasks (Cellstar) at a density of 1.17 x 10^6^ cells/flask 24h prior to transfection. The cells were transfected with 50 nM ASO 3 or NTC ASO using lipofectamine 2000. After 24h, cells were washed in 1x phosphate buffer saline (PBS) and subsequently incubated with puromycin containing media (InvivoGen, San Diego, California, USA, 10 µg/ml) for 10 min. Puromycin incorporation is a proxy for the mRNA translation rate *in vitro* and was measured by western blotting using an anti-puromycin antibody (MABE343, clone 12D10, Merck Millipore, Burlington, Massachusetts, USA, 1:25 000). The antibody was diluted in Milk/TBST (5% non-fat dry milk in TBS with 0.1% Tween20). A ponceau S staining (Sigma Aldrich, Saint Louis, Missouri, USA) was performed to verify equal loading. This assay was repeated three times to obtain three independent biological replicates.

**Mitochondrial and cytosolic fractionation**

3 x 10^6^ cells were seeded per condition in T175 culture flasks (Cellstar) 24h prior to transfection with 100 nM ASO 3 or NTC ASO using lipofectamine. After 24h, cells were treated for 10 min with puromycin containing media, as described before. Next, cells were rinsed twice using wash buffer (Tris 3.5 mM, NaCl 140 mM, pH 7.4), followed by a 5-10 min incubation step with harvest buffer (Tris 3.5 mM, NaCl 140 mM, 0.02% EDTA, pH 7.4). Cells were harvested and centrifuged twice at 500 g for 10 min. at 4 °C, followed by removal of the supernatant and cell pellet resuspension in cold wash buffer. Half of the suspension is used for mitochondrial fractionation, while the other half is centrifuged at 500 g for 10 min. at 4 °C, followed by removal of the supernatant and snap freezing. The latter part is used for cytosolic fractionation.

*Mitochondrial fractionation:* Mitochondrial fractions were prepared as described previously (Van Coster *et al*.^3^), with some minor adaptions. Briefly, the frozen cell pellets were homogenized in 19 volumes of buffer (10 mM Tris-HCl, 0.25 M sucrose, 2 mM EDTA, 50 U/mL heparin, pH 7.4) using a glass-glass pestle and sonicated shortly. Differential centrifugation in two steps was carried out. A first centrifugation at low speed (5600 g for 1 min) was performed to pellet cell debris, intact cells and nuclei. The resulting pellet was processed two times as described above and the combined supernatant was centrifuged at high speed (37 500 g for 3 min) to pellet the mitochondria and removal of other organelles. An additional washing step was performed. Finally, the resulting mitochondrial pellets were solubilized in a 1 % Triton-X 0.01 M, pH 7.5 PBS solution and protein concentrations were determined using the PierceTM modified Bradford Protein Assay kit (Thermo Scientific 23200, Thermo Fisher Scientific). The Triton-X mitochondrial isolates were immediately stored at -80 °C until use.

*Cytosolic fractionation:* Cells were centrifuged at 500 g for 5 minutes and the supernatant discarded. From the pelleted cells, cytoplasmic protein extracts were prepared using the NE-PER Nuclear and Cytoplasmic Extraction kit (Thermo Fisher Scientific), following the manufacturer’s instructions, with addition of protease inhibitors to the buffers (2 µg/ml leupeptin and aprotinin, 0.75 mM phenylmethylsulfonyl fluoride). Extracts were immediately stored at -80 °C until use.

Puromycin incorporation was measured by western blotting on both fractions, as described before. A ponceau S staining was performed to verify equal loading. Anti-beta-actin (Sigma Aldrich, A2228, 1:5000) and anti-SDHA (Abcam, Cambridge, United Kingdom, ab14715, 1:1000) antibodies were used to verify fractionation efficiency. The antibodies were diluted in Milk/TBST (5% non-fat dry milk in TBS with 0.1% Tween20). This assay was repeated three times to obtain three independent biological replicates.

**Western blot analysis**

Cells were lysed in RIPA lysis buffer (5 mg/ml sodium deoxycholate, 150 mM NaCl, 50 mM Tris-HCl pH 7.5, 0,1% SDS solution, 1% NP-40) supplemented with protease and/or phosphatase inhibitors. Protein concentrations were determined with the BCA protein assay (Bio-Rad, Hercules, California, USA). In total, 35 μg of protein lysate was loaded onto an SDS-PAGE gel (10% Pre-cast, Bio-Rad), ran for 1 h at 100 V and subsequently blotted onto a nitrocellulose membrane. HRP-labeled anti-rabbit (7074 S, Cell Signaling, Danvers, Massachusetts, USA, 1:10000 dilution) and anti-mouse (7076P2, Cell Signaling, 1:10000 dilution) antibodies were used as secondary antibodies. The antibodies were diluted in Milk/TBST (5% non-fat dry milk in TBS with 0,1% Tween20) and antibody binding was evaluated using the SuperSignal West Dura Extended Duration Substrate (Thermo Fisher Scientific) or SuperSignal West Femto Maximum Sensitivity Substrate (Thermo Fisher Scientific). Imaging was done using the Amersham Imager 680 (GE Healthcare, Chicago, Illinois, USA). Image J (version 1.52q) was used for the quantification of the blots. Uncropped scans of the blots can be found in Supplemental Fig 6.

**Reverse transcription quantitative polymerase chain reaction (RT-qPCR)**

Total RNA was extracted using the miRNeasy kit (Qiagen) according to the manufacturer’s instructions, including on-column DNase treatment. The Nanodrop (Thermo Fisher Scientific) was used to determine RNA concentrations and cDNA synthesis was performed using the iScript Advanced cDNA synthesis kit (Bio-Rad) using a mix containing 200 ng of RNA, 4 µl of 5x iScript advanced reaction buffer and 1 µl of iScript advanced reverse transcriptase. The qPCR reactions contain 2 µl of 1:4 diluted cDNA (2.5 ng/µl), 2.5 µl SsoAdvanced Universal SYBR Green Supermix (Bio-Rad), 0.25 µl forward (5 µM, IDT) and 0.25 µl reverse primer (5 µM, IDT) and was analyzed on a LC480 instrument (Roche).

For some purposes RNA was obtained using the SingleShot Cell Lysis Kit (Bio-Rad) according to the manufacturer’s instructions and cDNA synthesis was performed using the iScript Advanced cDNA synthesis kit (Bio-Rad) using a mix containing 8 µl sample lysate, 7 µl nuclease free water, 4 µl of 5x iScript advanced reaction buffer and 1 µl of iScript advanced reverse transcriptase. Subsequently, the cDNA is diluted 4 times prior to the qPCR reaction (see higher).

Expression levels were normalized using expression data of at least 2 stable reference genes out of 4 tested candidate reference genes (SDHA, HPRT1, UBC and TBP). Multi-gene normalization and relative quantification was performed using the qbase+ software (v3.2, www.qbaseplus.com).

The primer sequences used for qPCR were as follows:

*SAMMSON* Fw: CCTCTAGATGTGTAAGGGTAGT, Rv: TTGAGTTGCATAGTTGAGGAASDHA Fw: TGGGAACAAGAGGGCATCTG, Rv: CCACCACTGCATCAAATTCATG

HPRT1 Fw: TGACACTGGCAAAACAATGCA, Rv: GGTCCTTTTCACCAGCAAGCT

UBC Fw: ATTTGGGTCGCGGTTCTTG, Rv: TGCCTTGACATTCTCGATGGT

TBP Fw: CACGAACCACGGCACTGATT, Rv: TTTTCTTGCTGCCAGTCTGGAC

The MRPL4, MRPL13 and MRPL37 PrimePCR qPCR assays were purchased from Bio-Rad:

MRPL4 assay ID: qHsaCED0043059

MRPL13 assay ID: qHsaCED0004054

MRPL37 assay ID: qHsaCED0046878

**Quantitative PCR for evaluation of metastatic disease**

For assessment of metastatic disease in UM PDX models MEL077 and MP46, whole blood, lung and liver tissues were collected at the end of the experiment of ASO 3 or NTC ASO treated mice. Genomic DNA was isolated from liver and lung tissues (n=4 for MEL077 and n=6-7 for MP46 per treatment group) using the QIAamp DNA Mini Kit (Qiagen) and for blood (n=3-4 for MEL077 and n=6-7 for MP46 per treatment group) using the QIAamp DNA Blood Mini kit (Qiagen), according to the manufacturer’s instructions. The DropSense 96 (Trinean) was used to determine DNA concentrations. The qPCR reactions contain 2 µl of gDNA (6 ng/µl), 2.5 µl SsoAdvanced Universal SYBR Green Supermix (Bio-Rad), 0.25 µl forward (5 µM, IDT, Leuven, Belgium) and 0.25 µl reverse primer (5 µM, IDT) and was analysed on a LC480 instrument (Roche, Basel, Switzerland).

Copy number levels were determined for the human Alu-Sq, SVA and LINE-1 repetitive DNA sequences and murine gDNA assays located in the Hprt1 and Pthlh genes were used as reference genes for normalization. Analysis was performed using the qbase+ software (v3.2, www.qbaseplus.com). qPCR results from both PDX experiments were combined after log transformation, mean centering and autoscaling (according to Willems *et al.*^4^). For all human repetitive DNA sequence assays, high Cq values were obtained in the negative qPCR control. Five Cq values difference between the samples from the NTC ASO treated mice and the negative qPCR control was taken as a cutoff to conclude the presence of human DNA. Only lung samples fulfilled the criteria.

The oligonucleotide primers used for qPCR were as follows:

Alu-Sq Fw: CATGGTGAAACCCCGTCTCTA, Rv: GCCTCAGCCTCCCGAGTAG

SVA Fw: CTGTGTCCACTCAGGGTTAAAT, Rv: GAGGGAAGGTCAGCAGATAAAC

LINE-1 Fw: TGGCACATATACACCATGGAA, Rv: TGAGAATGATGGTTTCCAATTTC

Hprt1 Fw: CCTAAGATGAGCGCAAGTTGAA, Rv: CCACAGGACTAGAACACCTGCTAA

Pthlh: GACGTACAAAGAACAGCCACTCA, Rv: TTTTTCTCCTGTTCTCTGCGTTT

**RNA immunoprecipitation (RIP)**

80 x 10^6^ 92.1 and 100 x 10^6^ OMM1 cells were harvested from T75/T175 culture flasks (Cellstar) using trypsin, followed by a 5 min. centrifugation step at 500 g at 4 °C. After removal of the supernatant, cell pellets were resuspended in 1 ml cold PBS in 1.5 ml tube, followed by a second 5 min. centrifugation step at 600 g at 4 °C. After supernatant removal, pellets were flash frozen and stored at -80 °C until use. Pellets were lysed in 4 ml of polysome buffer (for 100 ml:  2 ml of TRIS 1 M pH 8.0, 4 ml of NaCl 5 M, 250 µl of MgCl2 50 µl of Triton 1 M and 100 µl of DTT 1 mM. Add fresh, 250 µl of RNAsin, (Promega), 1 ml of vanadyl ribonucleoside complexe solution and 2.5 ml of protease inhibitors, (Sigma Aldrich)) for 30 minutes on ice. Lysates were pre-cleaned with 20 µl of dynabeads (Thermo Fisher Scientific) per sample for 1 h at 4 °C with rotation. No antibody, 20 µg of human IgG (Abcam, ab2410), XRN2 (Bethyl laboratories, Montgomery, USA, A301-103A), p32 (Bethyl Laboratories, A302-863A) and MRPL13 antibody (Proteintech, Manchester, United Kingdom, 16241-1-AP) were added to lysates and incubated overnight at 4 °C.  Antibody-protein complexes were pulled down with 100 µl of rinsed beads per sample during 1 hour at 4 °C for 4 hours with rotation. Beads were captured on a magnetic rack and rinsed 5 times with polysome buffer. Rinsed beads were then resuspended in Qiazol (Qiagen) for RNA extraction following the manufacturer’s instructions. Finally, 1 µl of sequin spike (www.sequinstandards.com, 1:500 dilution) was added to 14 µl of RNA for cDNA synthesis with SsoAdvanced iScript (Bio-Rad). 10 µl of the undiluted RT product was added to 12.5 µl of Sso Advanced PreAmp Supermix and 2.5 µl of pre-amp primer pool for a pre-amplification run (98 °C - 3 min, 14 cycles of 98°C – 15 s, 58°C – 4 min). The pre-amplified cDNA was diluted 1000 times prior to qPCR as described before. *SAMMSON* Cq values were normalized to sequin Cq values using the qbase+ software (v3.2, www.qbaseplus.com). ​

The primer sequences used were as follows:

*SAMMSON* Fw: CCTCTAGATGTGTAAGGGTAGT, Rv: TTGAGTTGCATAGTTGAGGAA

Sequin Fw: ATGCTTTGATCGCGTTGGTG, Rv: AGCAAAACGAACGGACAATGA

**ChIRP-MS affinity purification**

75 x 10^6^ – 100 x 10^6^ cells were cultured in 145 cm^2^ dishes at a maximum confluency of 80%, washed once with ice-cold PBS, and UV cross-linked in ice-cold PBS at 254 nm to an accumulated energy intensity of 400 mJ/cm^2^. Cells were scraped in ice-cold PBS, and split equally among eight microcentrifuge tubes. ChIRP lysis buffer (20 mM Tris-HCl pH 7.5, 200 mM NaCl, 2.5 mM MgCl_2_, 0.05% NP-40, 0.1% SDS)^2^ was supplemented with fresh 0.1% sodiumdeoxycholate, 60 U/mL Superase-In Rnase inhibitor (Invitrogen, Carlsbad, California, USA), 1 mM DTT, 0.5 mM PMSF, and protease inhibitor cocktail (Roche). Cell pellets were resuspended in supplemented ChIRP lysis buffer, and sonicated with a Bioruptor (Diagenode, Seraing, Belgium) until lysates appeared clear. 10% of the ChIRP sample was used for RNA extraction of input material. Thereafter, 6.23 µl of 50 µM *SAMMSON* or LacZ biotinylated capture probes (LGC Biosearch Technologies, Novato, California, USA) were bound to 100 µl of equilibrated Rnase-free Dyna-One C1 magnetic beads (Thermo Fisher Scientific) per sample and were incubated overnight at 4 °C with end-to-end rotation. Next day, *SAMMSON* or LacZ probe-bound beads were added to the lysates and lysates were rotated for 3 h at 4 °C. Bead-bound fractions were washed three times with unsupplemented ChIRP lysis buffer. 10% of the sample was used for RNA extraction to validate RNA pulldown on RT-qPCR. Next, beads were washed three times with Rnase-free trypsin digestion buffer (20 mM Tris-HCl pH 7.5, 2 mM CaCl_2_), and were ultimately resuspended in 20 µl 20 mM Tris-HCl pH 7.5. 750 ng trypsin was added directly on the beads, and digestion was left overnight at 37 °C. Next day, an additional 250 ng trypsin was added and incubated for 3 h at 37 °C. Peptides were acidified to a final concentration of 2% formic acid. All experiments were performed in biological triplicates for label-free quantitative proteomic analysis.

**LC-MS/MS instrument analysis**

Peptide mixtures were run on an Ultimate 3000 RSLC nano LC (Thermo Fisher Scientific) connected in-line to a Q-Exactive HF mass spectrometer (Thermo Fisher Scientific). In brief, peptides were loaded on an in-house made trapping column (100 µm i.d. x 20 mm, 5 µM C18 Reprosil-HD beads, Dr. Maisch, Ammerbuch-Entringen, Germany). After flushing the trapping column, peptides were loaded in solvent A (0.1% formic acid) on an in-house made reverse-phase column (75 µm i.d. x 250 mm, 3 µm Reprosil-Pur-basic-C18-HD beads packed in the needle, Dr. Maisch, Ammerbuch-Entringen, Germany) and eluted by a linear gradient of solvent B (0.1% formic acid in acetonitrile) from 2% to 55% in 1.5 h, and subsequently washed with 99% solvent B. All steps were run at a constant flow rate of 300 nl/min. The mass spectrometer was operated in a data-dependent acquisition, positive ionization mode, automatically switching between MS and MS/MS acquisition for the five most abundant peaks in a MS spectrum. Source voltage was 3.4 kV, and capillary temperature was 275 °C. One MS1 scan (m/z 400-2000, AGC target 3 x 10^6^ ions, maximum ion injection time 80 ms), acquired at a resolution of 70 000 (at 200 m/z), was followed by up to five tandem MS scans (resolution 17 500 at 200 m/z) of the most intense ions, fulfilling the predefined selection criteria (AGC target 5 x 104 ions, maximum ion injection time 80 ms, isolation window 2 Da, fixed first mass 140 m/z, spectrum data type: centroid underfill ratio 2%, intensity threshold 1.3 x 104, exclusion of unassigned 1, 5 - 8, and >8 positively charged precursors, peptide match preferred, exclude isotopes on, dynamic exclusion time 12 ms). The HCD collision energy was set to 25% normalized collision energy, and the poly(dimethylcyclosiloxane) background ion at 445.120025 Da was used for internal calibration (lock mass).

**MaxQuant and Perseus MS data processing and analysis**

Xcalibur raw files were analysed using the Andromeda search engine implemented in MaxQuant (MaxQuant v1.6.0.1). Spectra were searched against the human UniProt sequence database. Methionine oxidation and N-terminal acetylation were set as variable modifications. The minimum label-free quantitation ratio count was 2, and the Fast LFQ option was disabled. After the searches were completed, LFQ intensities were imported in Perseus (v1.5.8.5) for downstream analysis.

LFQ intensities were log 2 transformed, and contaminant proteins, reverse hits, and protein only identified by site were excluded from the analysis. Three valid values in at least one sample group (i.e. pulldown of *SAMMSON* or LacZ) was used for a protein to be included in further analysis. Missing values were imputed from a normal distribution of intensities. A two-sided t-test (0.05 FDR, 1000 randomizations) was performed to identify differential proteins in volcano plots. The default S0-value (0.1) in Perseus was maintained for generating the S-curve in the volcano plots. The mass spectrometry proteomics data have been deposited to the ProteomeXchange Consortium via the PRIDE^5^ partner repository with the dataset identifier PXD023511. Reactome (https//reactome.org/) was used to perform overrepresentation analysis.

**JC-1 fluorescent staining**

Cells were seeded in Nunc Lab-Tek Chamber slides at a density of 100 000 cells/well 24 h prior to transfection. Cells were transfected with 100 nM ASO 3 or NTC ASO using lipofectamine 2000. Cells were stained with 5 μg/ml of 5,5′,6,6′-tetraethylbenzimidazolyl-carbocyanine iodide (JC-1; Invitrogen) for 30 min at 37 °C following a published procedure^6^. Live cells were visualized under a fluorescence microscope (Olympus, Hamburg, Germany), detecting red and green fluorescent emission separately using optical filters. Red over green JC-1 fluorescence ratios were determined by converting images to bright field and measuring the average grayscale (Cell F software; Olympus) in ten microscopic fields ×400 magnification selected at random, and reported as mean values ± SD.

**Seahorse XF Cell Mito Stress Test**

A seahorse XF Cell Mito Stress Test was performed to measure the oxygen consumption rate (OCR). Cells were seeded in T25 or T75 culture flasks (Cellstar) at a density of 390 000 cells (T25) or 1 170 000 cells (T75) 24 h prior to transfection. The cells were transfected with 100 nM ASO 3, NTC ASO, 100 nM MRPL4, MRPL13 and MRPL37 siPOOLs or NTC siPOOLs using lipofectamine 2000. At least three replicates were included in every experiment. Four hours later, 15 000 cells were transferred to Seahorse XFp Cell Culture Miniplates (Agilent Technologies, Santa Carla, California, USA) and were allowed to settle overnight (ASO 3 and NTC ASO treatment) or were incubated for 72h (MRPL4, MRPL13 and MRPL37 siPOOLs and NTC siPOOLs treatment). Subsequently, oxygen consumption rates were measured in triplicates for each condition using the Seahorse XFp device (Agilent Technologies) according to the standard mito stress test procedures in seahorse assay medium supplemented with 14.3 mM glucose, 1 mM pyruvate and 2 mM glutamine (Sigma Aldrich), and cells were sequentially challenged with 1 µM oligomycin, 0.5 µM (92.1) or 1 µM (OMM2.3 and OMM1) carbonyl cyanide 4-(trifluoromethoxy)-phenylhydrazone (FCCP) and 0.5 µM of a rotenone antimycin A mix (Agilent Technologies). Following the assay, protein concentrations were calculated based upon absorbance reading at 280 nm (Biodrop, Isogen Life Science, Utrecht, The Netherlands) for normalization of the results. Spare respiratory capacity was calculated as the difference between maximal and the basal oxygen consumption rate. Relative oxygen consumption rates are relative compared to NTC ASO values.

**Differential gene expression analysis by RNA sequencing**

RNA sequencing was performed on quadruplicates of NTC ASO or ASO 3 (50 nM) treated 92.1 and OMM1 cells. Libraries for RNA sequencing were prepared using the Quant Seq 3’ end library prep according to the manufacturer’s instructions (Lexogen, Vienna, Austria) (2.5 µl input of RNA lysates) and quantified on a Qubit Fluorometer prior to single-end sequencing with 75 bp read length on a NextSeq 500 sequencer (Illumina, San Diego, California, USA).

RNA sequencing was performed on UM PDX MEL-077 tumor samples treated with either NTC ASO or ASO 3 (10 mg/kg, 3.5x per week, n=4/treatment group) for 3 weeks. Libraries for RNA sequencing were prepared using the Truseq mRNA library prep according to the manufacturer’s instructions (Illumina) (500 ng input of purified RNA) and quantified on a Qubit Fluorometer prior to single-end sequencing with 75 bp read length on a NextSeq 500 sequencer (Illumina). Reads were mapped to the human genome (hg38) using STAR and gene expression was quantified using HTSeq (v0.6.1). Differentially expressed genes were identified using DESeq2 (v1.26.0). Pre-ranked gene set enrichment analysis (GSEA 4.1.0) was performed using c2.all.v7.2.symbols (curated gene sets), h.all.v7.2.symbols (Hallmark gene sets) and c6.all.v7.2.symbols (oncogenic signature gene sets) (Molecular Signatures Database (MsigDB)), applying 1000 permutations and a classic enrichment statistic.

Primary and metastatic UM samples were histologically reviewed by a pathologist before nucleic acids extraction in order to select samples with at least 30% of tumor cells. RNA was extracted using TRIzol (Qiagen) and subsequently purified on Zymo-Spin™ IC (Zymo Research, Irvine, CA, USA). RNA was quantified by Nanodrop (Thermo Fisher Scientific) and RNA integrities were assessed by BioAnalyzer 2100 (Agilent Technologies). RNA Sequencing (RNAseq) libraries were prepared using the TruSeq Stranded mRNA kit (Illumina) from 1 µg RNA, isolated from initial libraries with median insert size of 300 bp according to the manufacturers’ protocols. Libraries were 100 bp paired-end multiplex sequenced on the Illumina HiSeq 2500 (Illumina).  Raw sequencing data were aligned by STAR with hg38 as the reference genome and GENCODE v22 as gene annotation. Gene abundances were estimated by RSEM^7^.

**Immunohistochemistry (IHC)**

Tumor samples of UM PDX mice (MEL077) that have been treated with ASO 3 or NTC ASO (10 mg/kg, 3.5x per week, n=4/treatment group) for 3 weeks were fixed in 4% formaldehyde and subsequently embedded in paraffin to obtain formalin-fixed, paraffin-embedded (FFPE) samples. Tumor sections were used for hematoxilin and eosin (HE) staining, and for immunohistochemistry with antibodies against Ki-67 (RTU, clone 30-9, Roche) and caspase-3 (R&D Systems, Minneapolis, Minnesota, USA, AF835, 1:2000) on a Ventana Benchmark Ultra automated staining system (Roche). The presence of apoptotic bodies was semi-quantitatively scored by two independent pathologists, blinded to treatment allocation, as follows: not present (-), rare (+), easily recognized (++), abundant (+++). Based on these scores, a diagnosis was made as ‘ASO 3-treated’ or ‘NTC ASO-treated’ for each tumor sample. Ki-67 expression was assessed by identifying areas with the most intense nuclear staining (‘hot spots’) at low magnification (100x). The percentage of immunoreactive tumor cells was calculated by counting at least 500 cells within each hot spot. Additionally, the mitotic rate was determined by counting the number of cells in the M-phase in 10 random high-power fields (400x) on H&E sections.

**Statistical analysis**

Statistical analyses and data visualizations were performed with Graphpad Prism version 9.0.0 (GraphPad Software, San Diego, California, USA). The individual data points and mean or mean ± s.d. were presented, unless otherwise specified. Significance between treatment groups was determined using a one-tailed or two-tailed Student’s t-test when comparisons are made between two groups. For comparisons between more than two groups, one-way ANOVA or two-way ANOVA was used with multiple testing correction. Significant differences between FPKMs and TPMs (RNA sequencing data) was determined using the Mann-Whitney test. Significant differences between *SAMMSON* TPMs of matching primary and metastatic UM tumor samples was determined using the Wilcoxon matched-pairs signed rank test. The level of statistical significance was set at p<0.05 (* p≤0.05, ** p≤0.01, *** p≤0.001, **** p≤0.0001).

References

1 Vendramin R, Konnova A, Adnane S, Cinque S, Katopodi V, Knezevic Z *et al.* Activation of the Integrated Stress Response in drug-tolerant melanoma cells confers vulnerability to mitoribosome-targeting antibiotics. bioRxiv 2020; 58: 50–52.

2 Leucci E, Vendramin R, Spinazzi M, Laurette P, Fiers M, Wouters J *et al.* Melanoma addiction to the long non-coding RNA SAMMSON. Nature 2016; 531: 518–522.

3 van Coster R, Smet J, George E, de Meirleir L, Seneca S, van Hove J *et al.* Blue native polyacrylamide gel electrophoresis: A powerful tool in diagnosis of oxidative phosphorylation defects. Pediatric Research 2001; 50: 658–665.

4 Willems E, Leyns L, Vandesompele J. Standardization of real-time PCR gene expression data from independent biological replicates. Analytical Biochemistry 2008; 379: 127–129.

5 Perez-Riverol Y, Csordas A, Bai J, Bernal-Llinares M, Hewapathirana S, Kundu DJ *et al.* The PRIDE database and related tools and resources in 2019: improving support for quantification data. Nucleic acids research 2019; 47: D442–D450.

6 de Paepe B, Smet J, Vanlander A, Seneca S, Lissens W, de Meirleir L *et al.* Fluorescence imaging of mitochondria in cultured skin fibroblasts: a useful method for the detection of oxidative phosphorylation defects. Pediatric Research 2012; 72: 232–240.

7 Li B, Dewey CN. RSEM: Accurate transcript quantification from RNA-Seq data with or without a reference genome. BMC Bioinformatics 2011; 12: 1–16.
