# Supplementary figures and images for: The long non-coding RNA SAMMSON is essential for uveal melanoma cell survival
Source: Oncogene. 2021 Sep 10;41(1):15–25. doi: 10.1038/s41388-021-02006-x (PMC8724009; doi:10.1038/s41388-021-02006-x)

## Vital status

SAMMSON  
expression

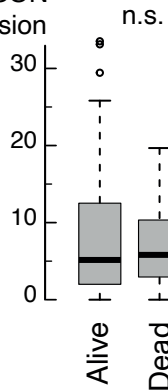

## Clinical stage

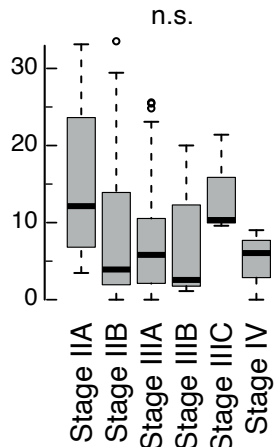

## Primary tumor site

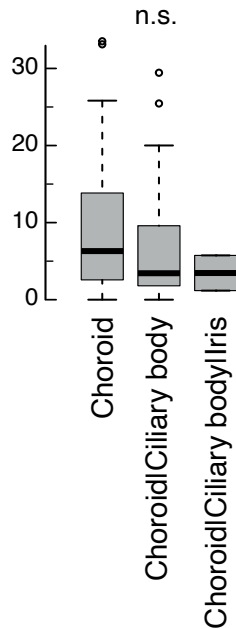

## Metastatic stage

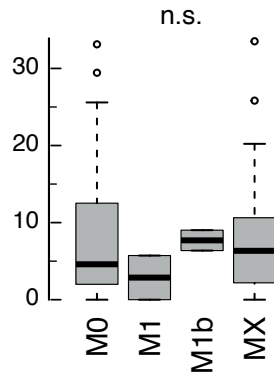

Supplement: Supplementary file 3 — Supplemental Fig 1. SAMMSON expression is independent from patient survival, tumor stage, tumor localization site and metastatic state of the UM patient. [file 41388_2021_2006_MOESM3_ESM.pdf]

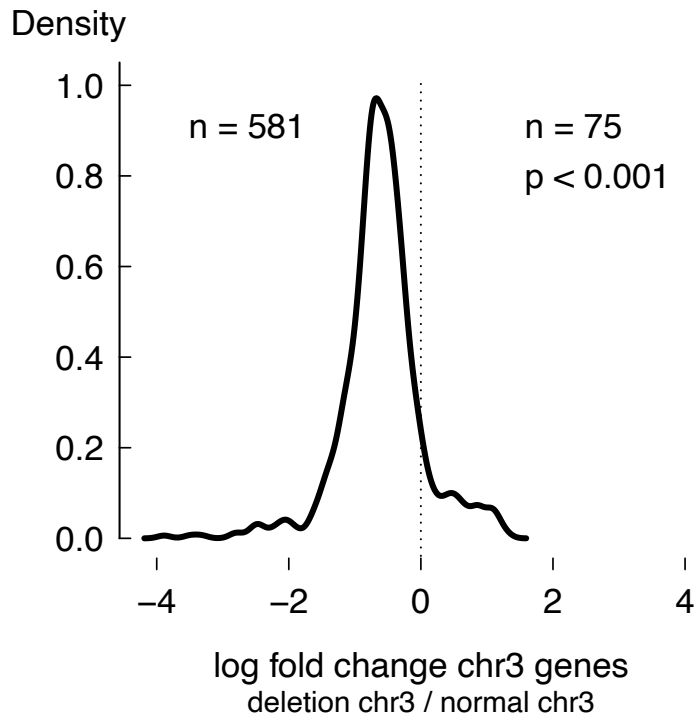

Supplement: Supplementary file 4 — Supplemental Fig 2. Expressed genes in monosomy 3 UM tumors compared to disomy 3 UM tumors. [file 41388_2021_2006_MOESM4_ESM.pdf]

A

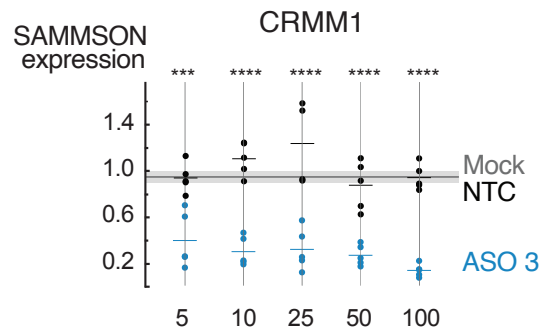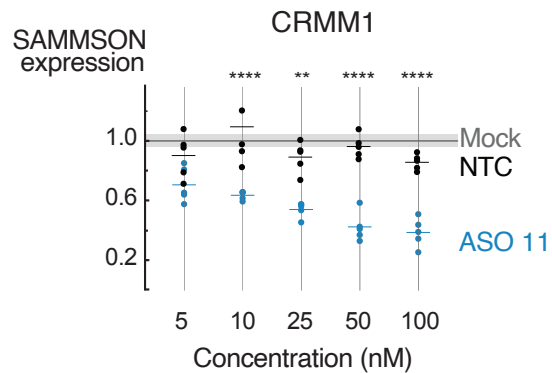

B

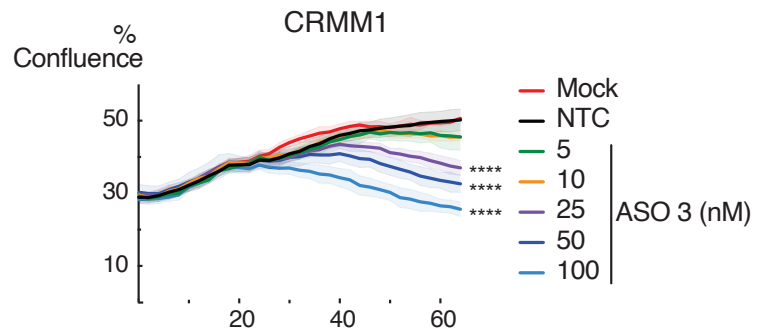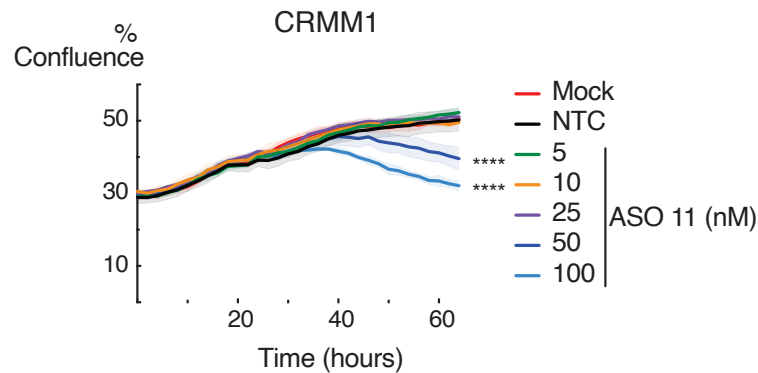

Supplement: Supplementary file 5 — Supplemental Fig 3. SAMMSON knockdown affects cell growth in CM cell line CRMM1. [file 41388_2021_2006_MOESM5_ESM.pdf]

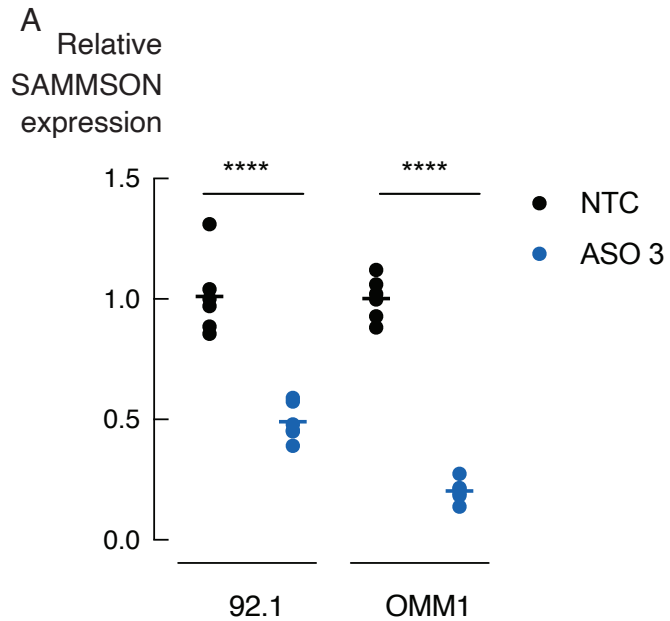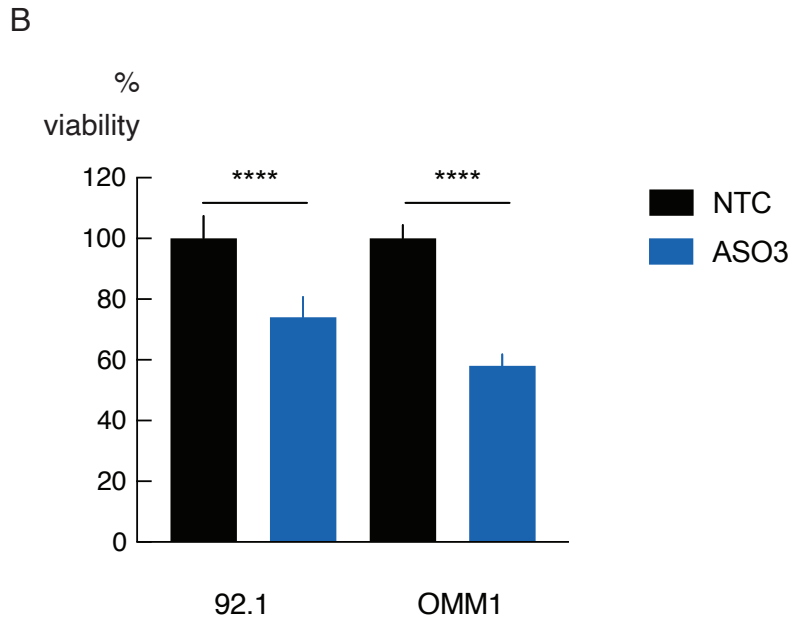

Supplement: Supplementary file 6 — Supplemental Fig 4. Non-lipid-based delivery of ASOs in UM cells. [file 41388_2021_2006_MOESM6_ESM.pdf]

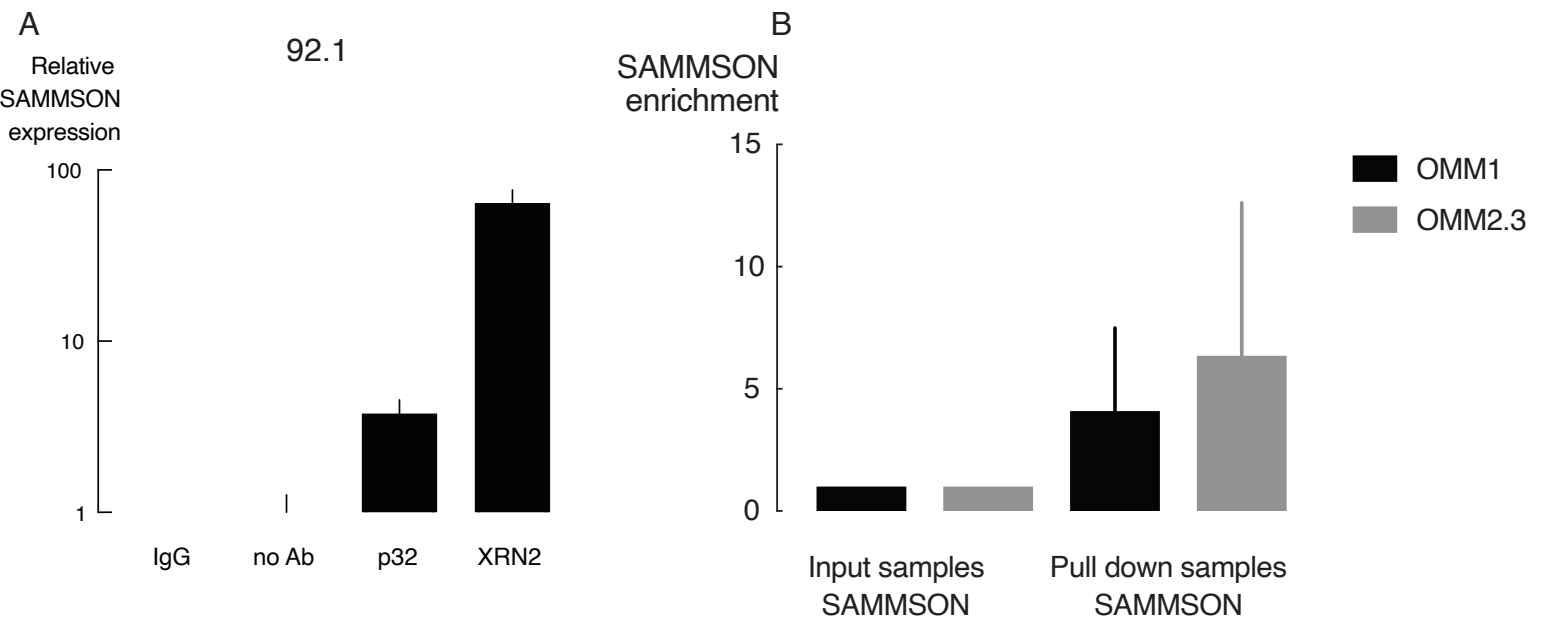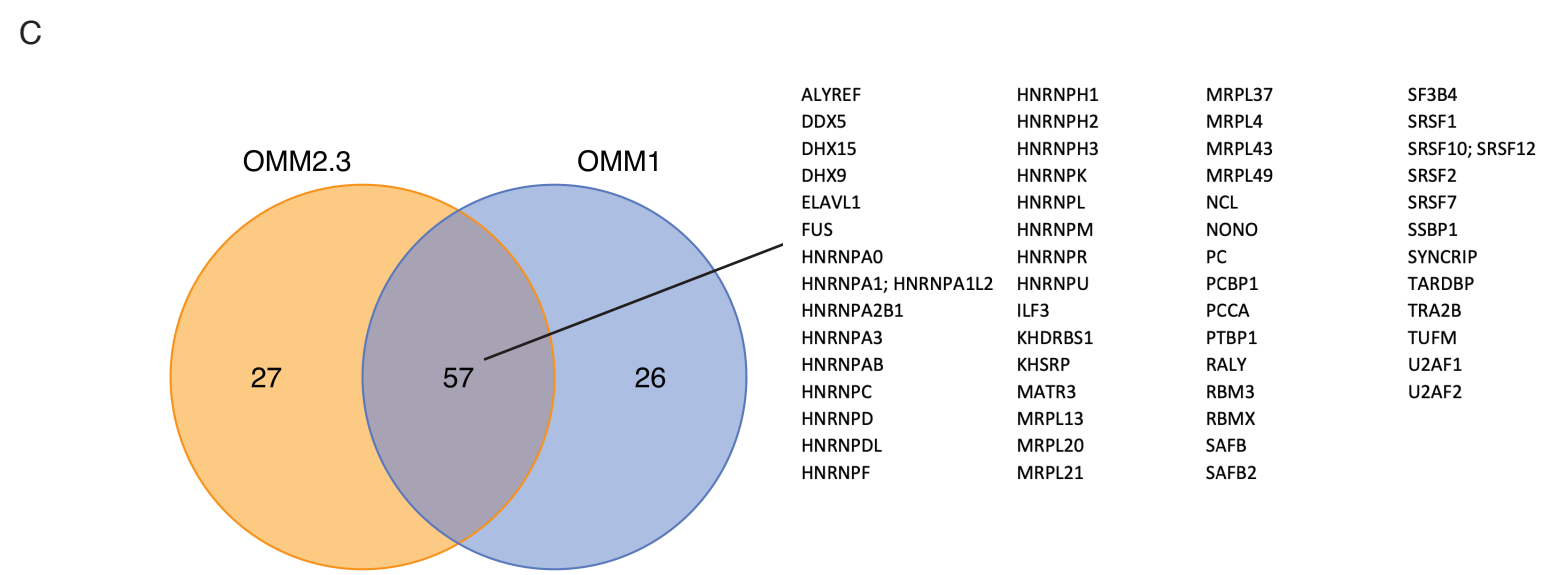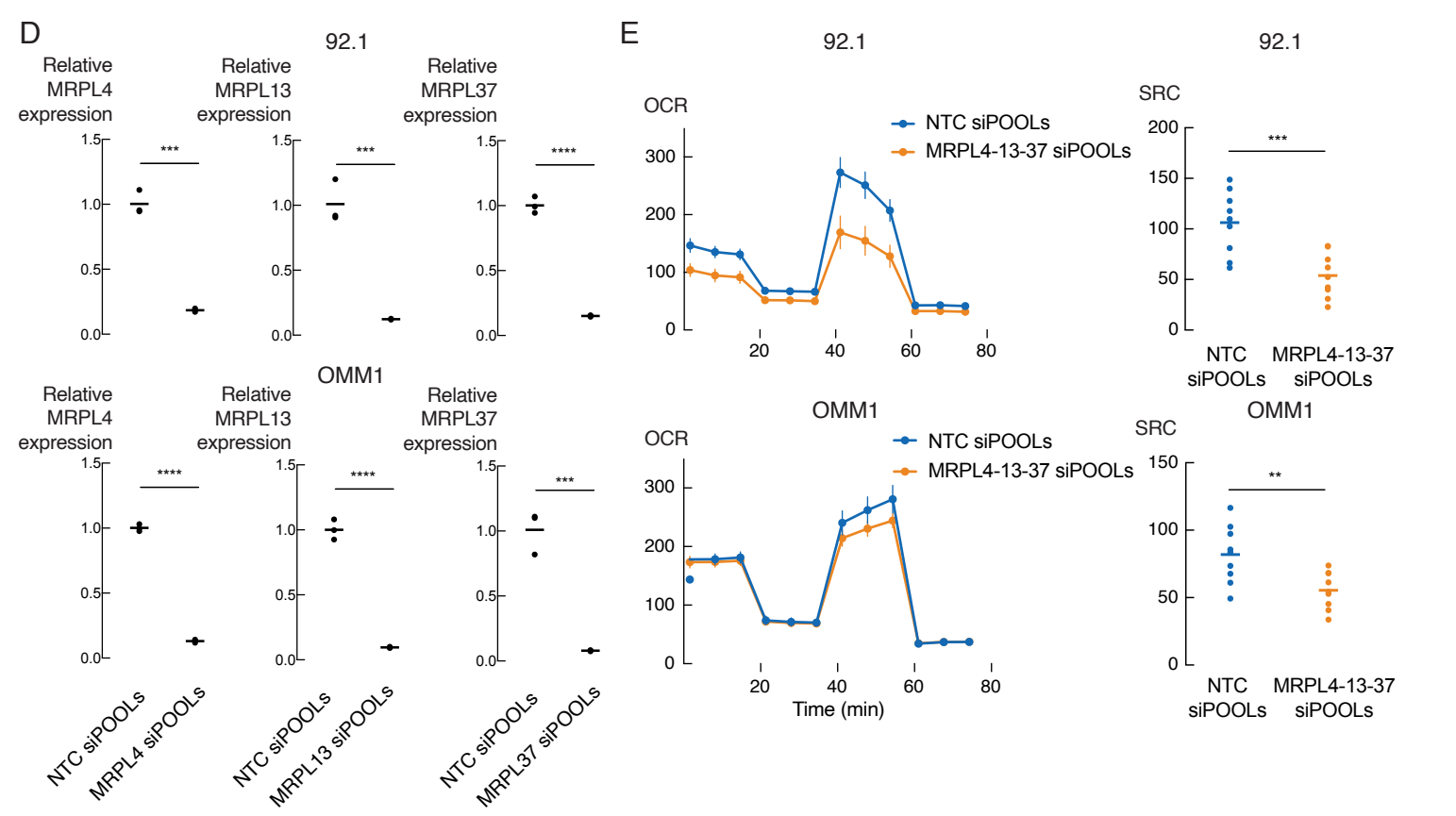

Supplement: Supplementary file 7 — Supplemental Fig 5. Identification of SAMMSON interaction partners. [file 41388_2021_2006_MOESM7_ESM.pdf]

A

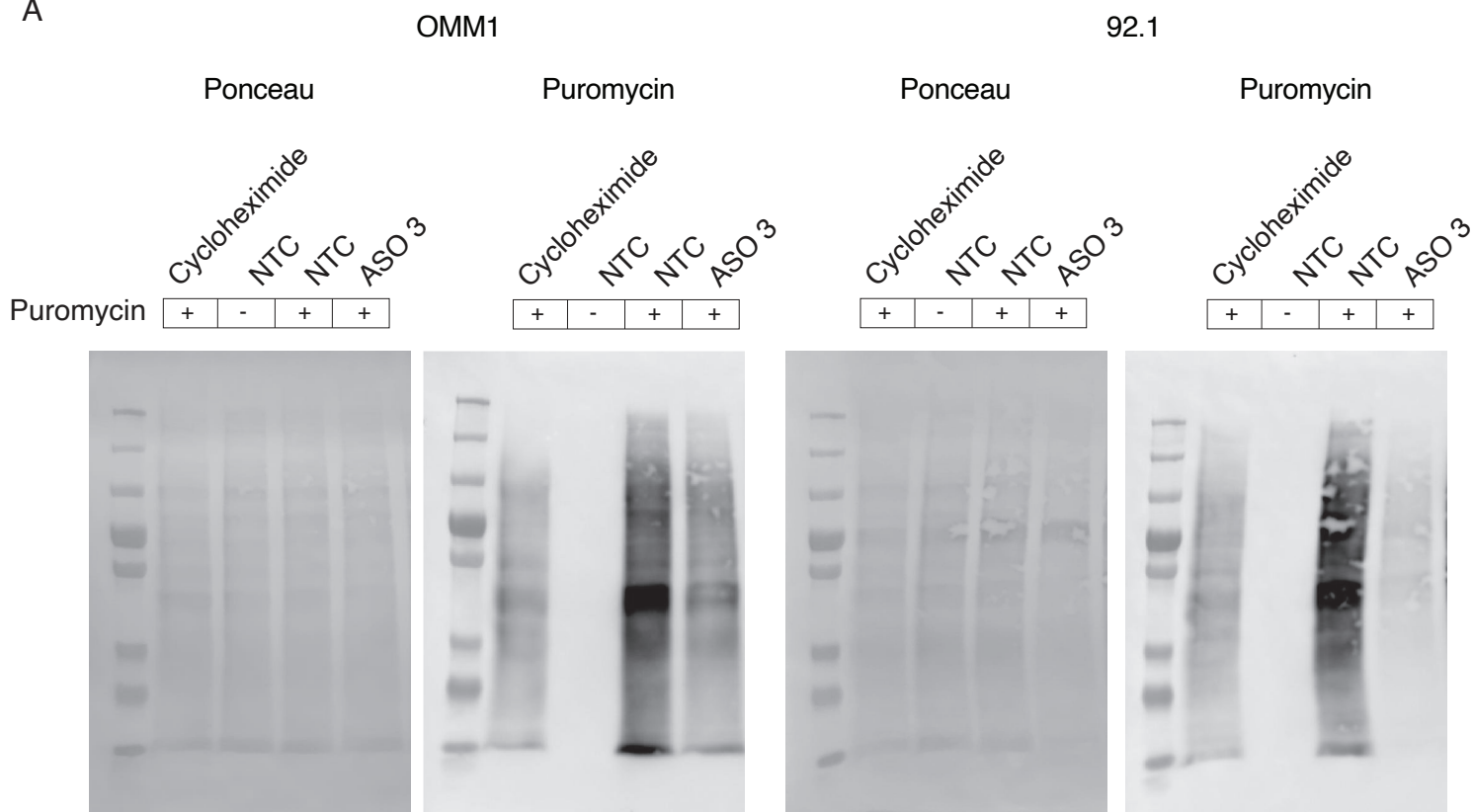

B

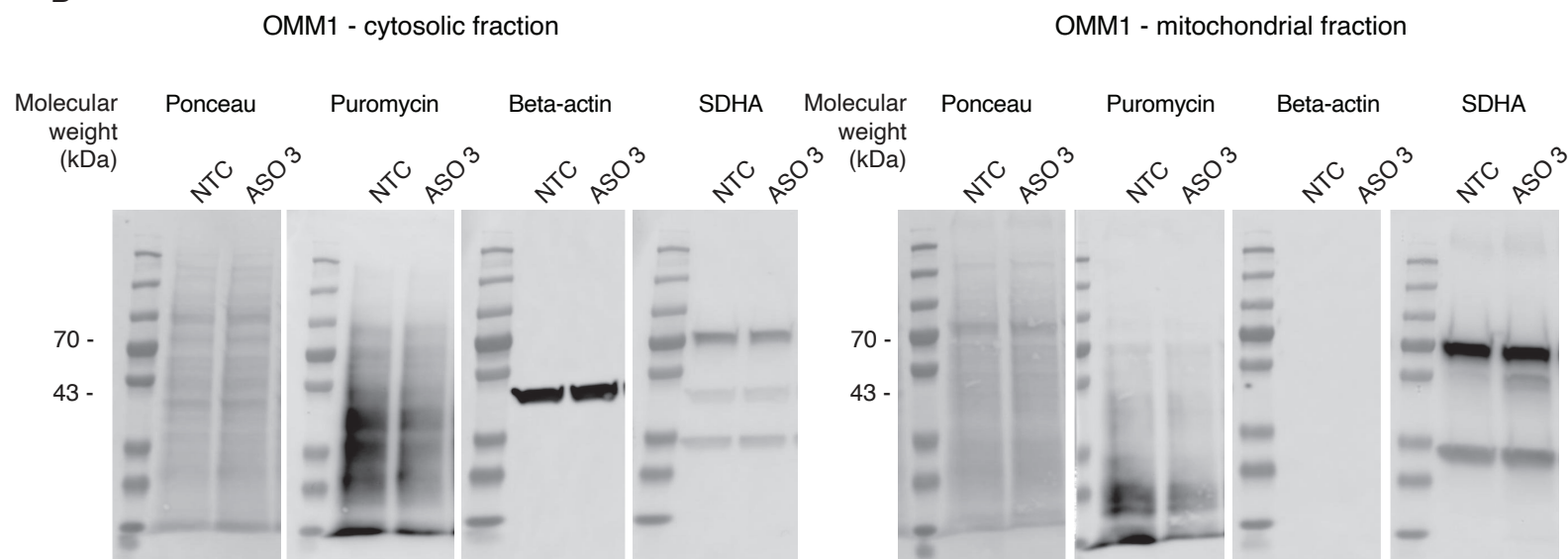

Supplement: Supplementary file 8 — Supplemental Fig 6. Uncropped images of WB-SUnSET analysis. [file 41388_2021_2006_MOESM8_ESM.pdf]

A

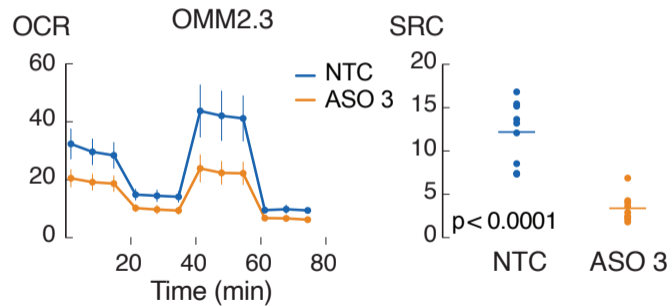

B

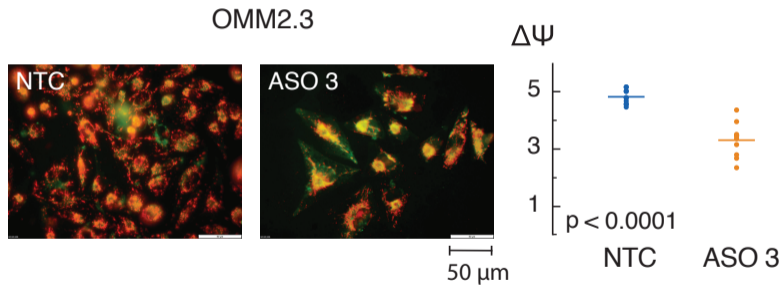

Supplement: Supplementary file 9 — Supplemental Fig 7. Impairment of mitochondrial function upon SAMMSON knockdown in UM cell line OMM2.3. [file 41388_2021_2006_MOESM9_ESM.pdf]

A

Normalized  
confluence

92.1

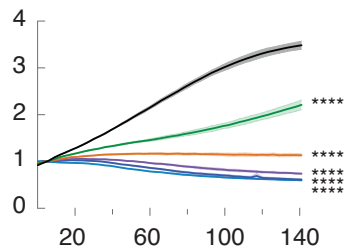

OMM1

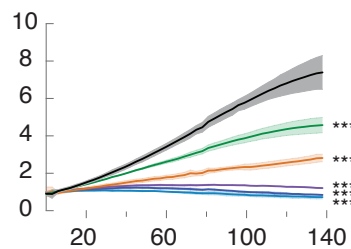Relative  
Annexin V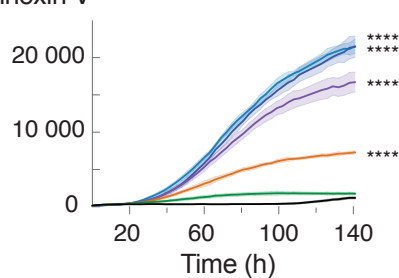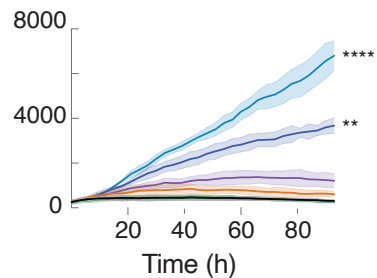

— 0 — 3.125 — 6.25 — 12.5 — 25 — 50

Tigecycline ( $\mu\text{M}$ )

B

Relative %  
confluence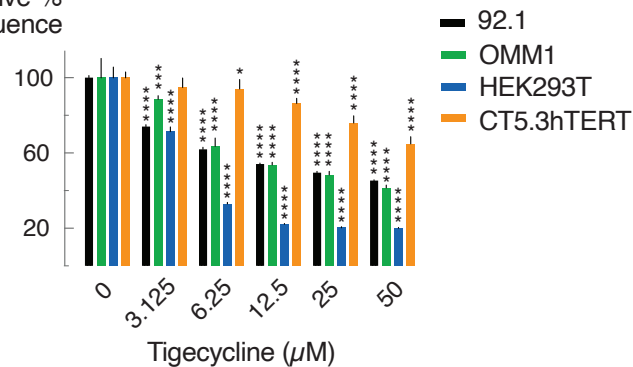

Supplement: Supplementary file 10 — Supplemental Fig 8. Phenotypic results using tigecycline are comparable to SAMMSON inhibition in UM cells. [file 41388_2021_2006_MOESM10_ESM.pdf]

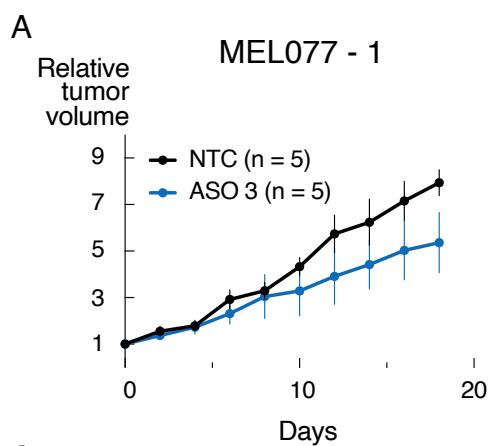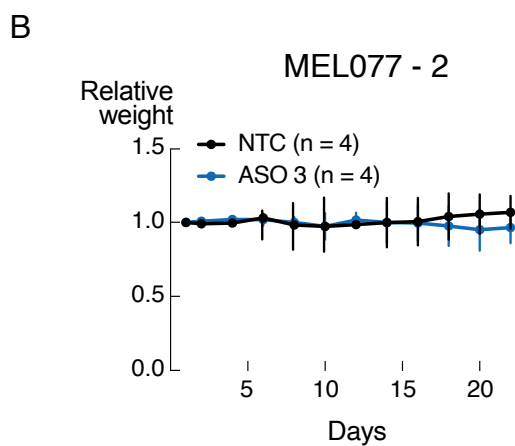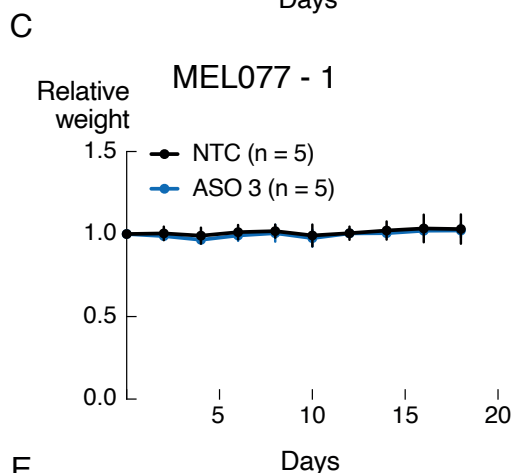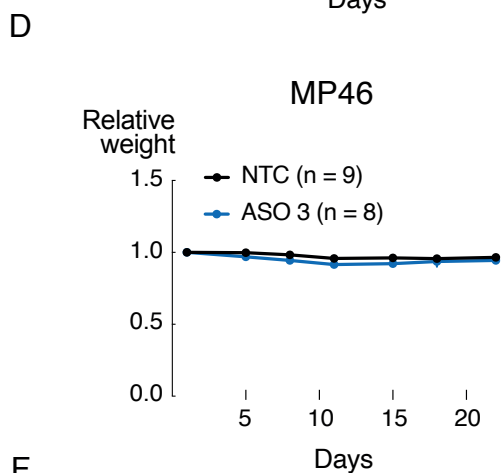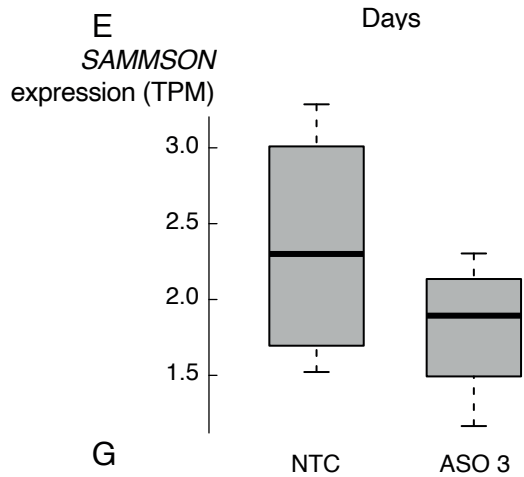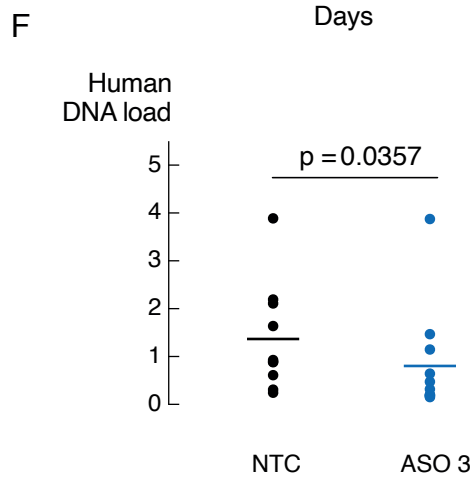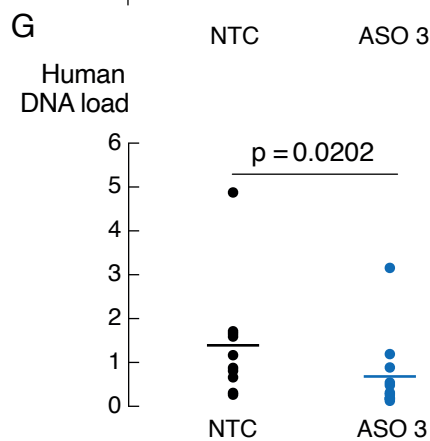

Supplement: Supplementary file 11 — Supplemental Fig 9. SAMMSON inhibition slows down tumor growth in vivo. [file 41388_2021_2006_MOESM11_ESM.pdf]
